# Supplementary material for: The evolution of the temporal program of genome replication
Source: Nat Commun. 2018 Jun 6;9:2199. doi: 10.1038/s41467-018-04628-4 (PMC5989221; doi:10.1038/s41467-018-04628-4)
Supplement: Supplementary file 1 — Supplementary Information [file 41467_2018_4628_MOESM1_ESM.pdf]

Supplementary Figures

The evolution of the temporal program of genome replication

Agier et al.

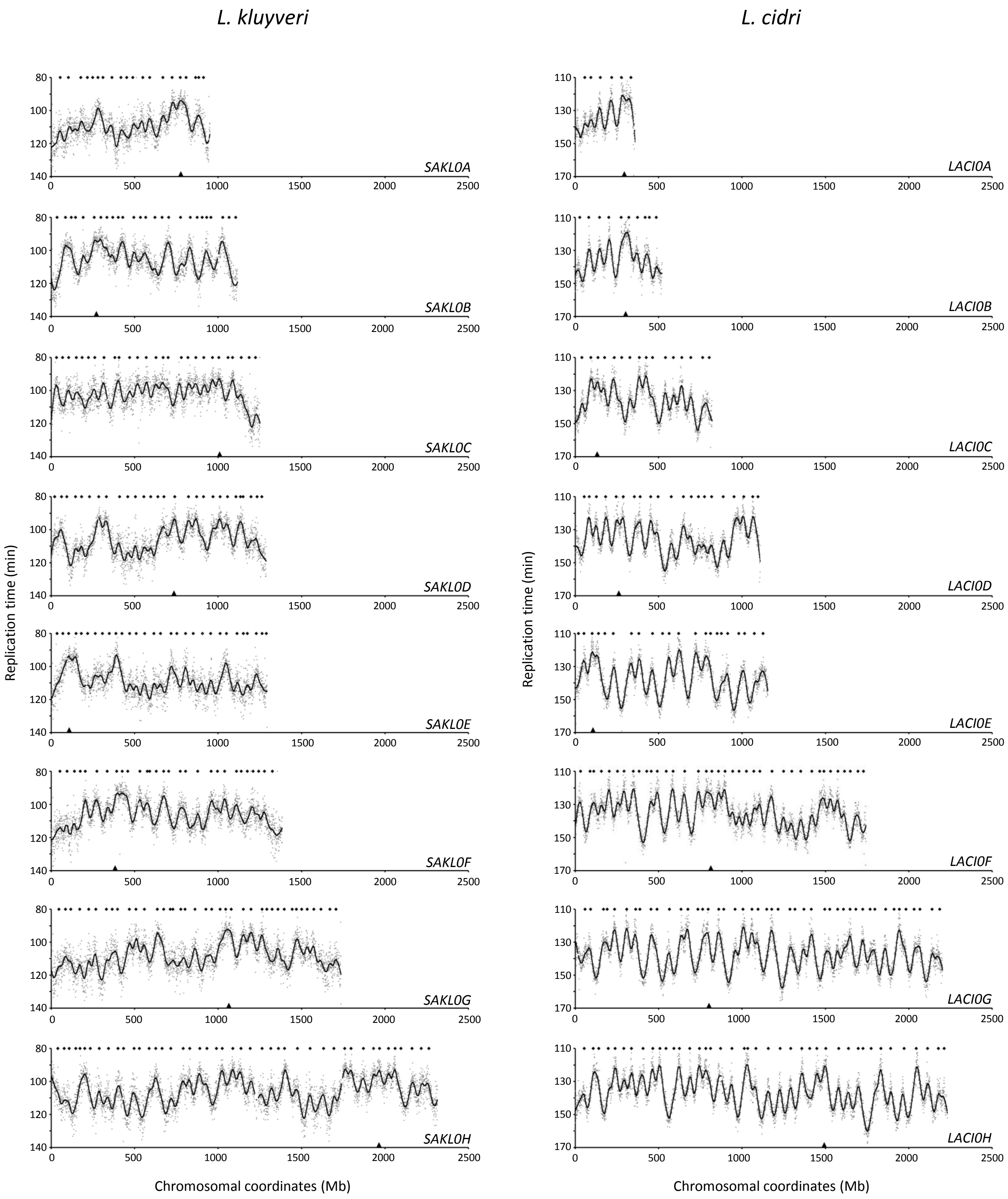
L. kluyveri
L. cidri
SAKL0A
SAKL0B
SAKL0C
SAKL0D
SAKL0E
SAKL0F
SAKL0G
SAKL0H
LACI0A
LACI0B
LACI0C
LACI0D
LACI0E
LACI0F
LACI0G
LACI0H
Replication time (min)
Chromosomal coordinates (Mb)

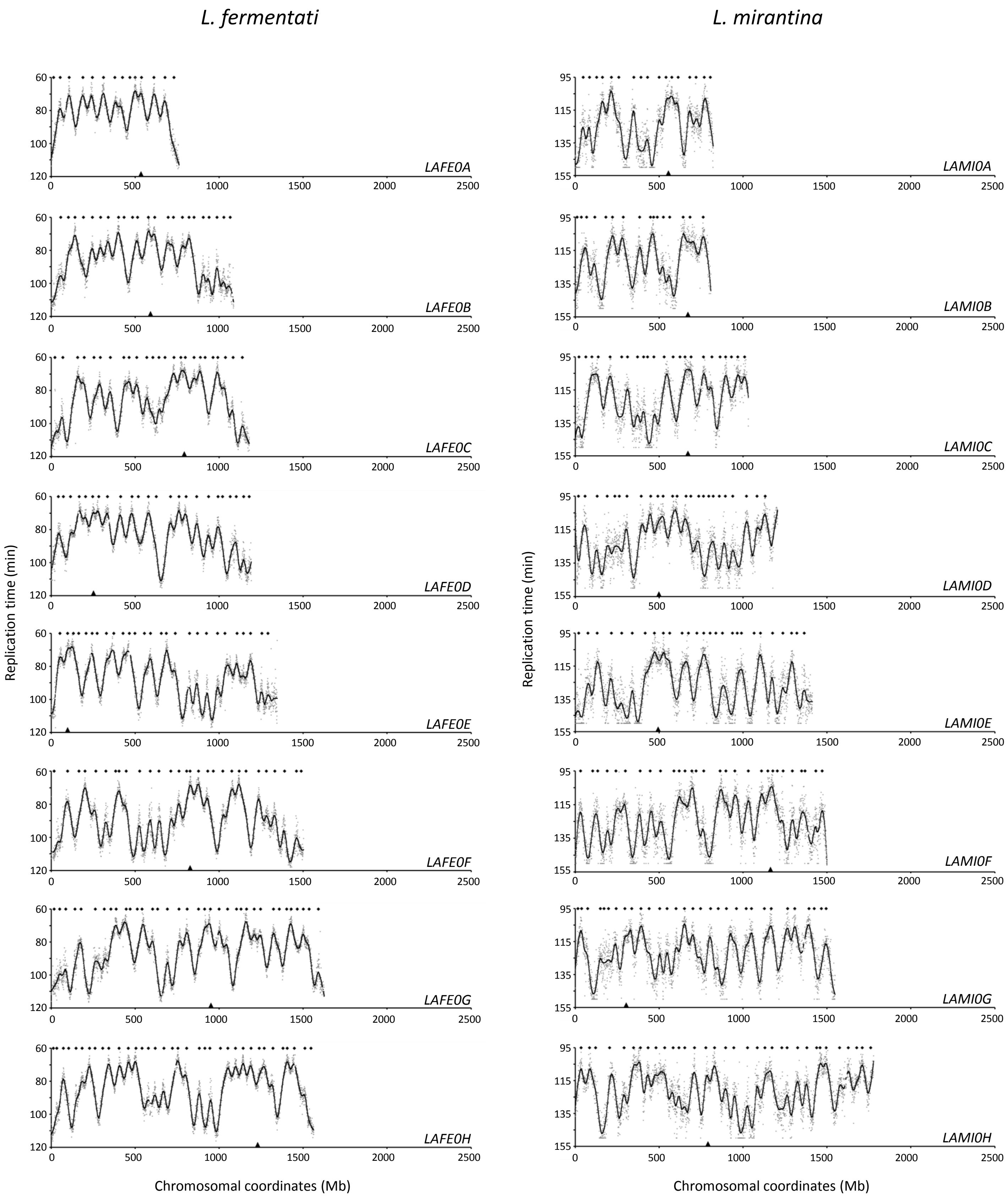

L. fermentati
L. mirantina
LAFE0A
LAFE0B
LAFE0C
LAFE0D
LAFE0E
LAFE0F
LAFE0G
LAFE0H
LAMI0A
LAMI0B
LAMI0C
LAMI0D
LAMI0E
LAMI0F
LAMI0G
LAMI0H
Replication time (min)
Chromosomal coordinates (Mb)

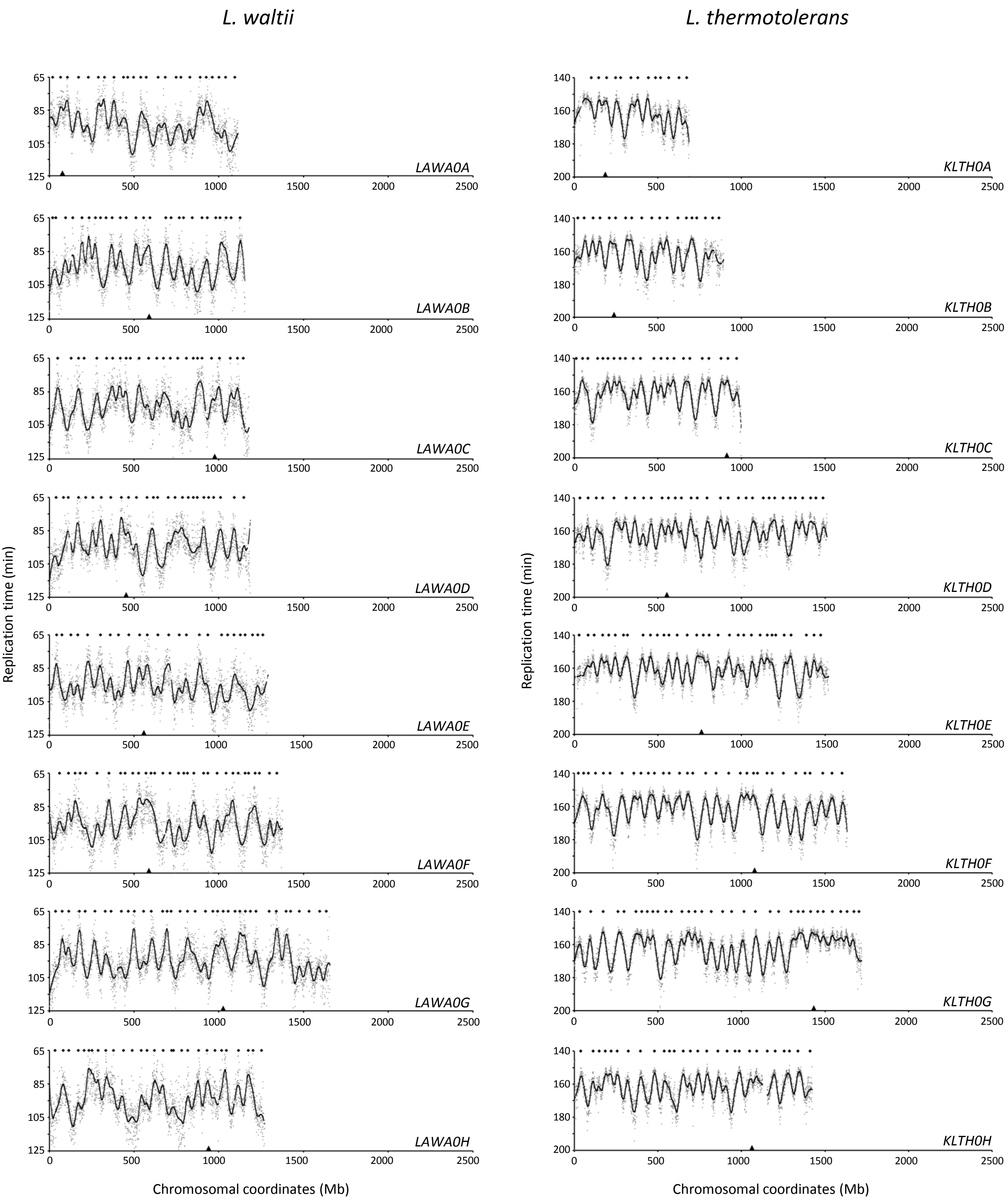
L. waltii
L. thermotolerans
Replication time (min)
Replication time (min)
LAWA0A
LAWA0B
LAWA0C
LAWA0D
LAWA0E
LAWA0F
LAWA0G
LAWA0H
KLTH0A
KLTH0B
KLTH0C
KLTH0D
KLTH0E
KLTH0F
KLTH0G
KLTH0H
Chromosomal coordinates (Mb)
Chromosomal coordinates (Mb)

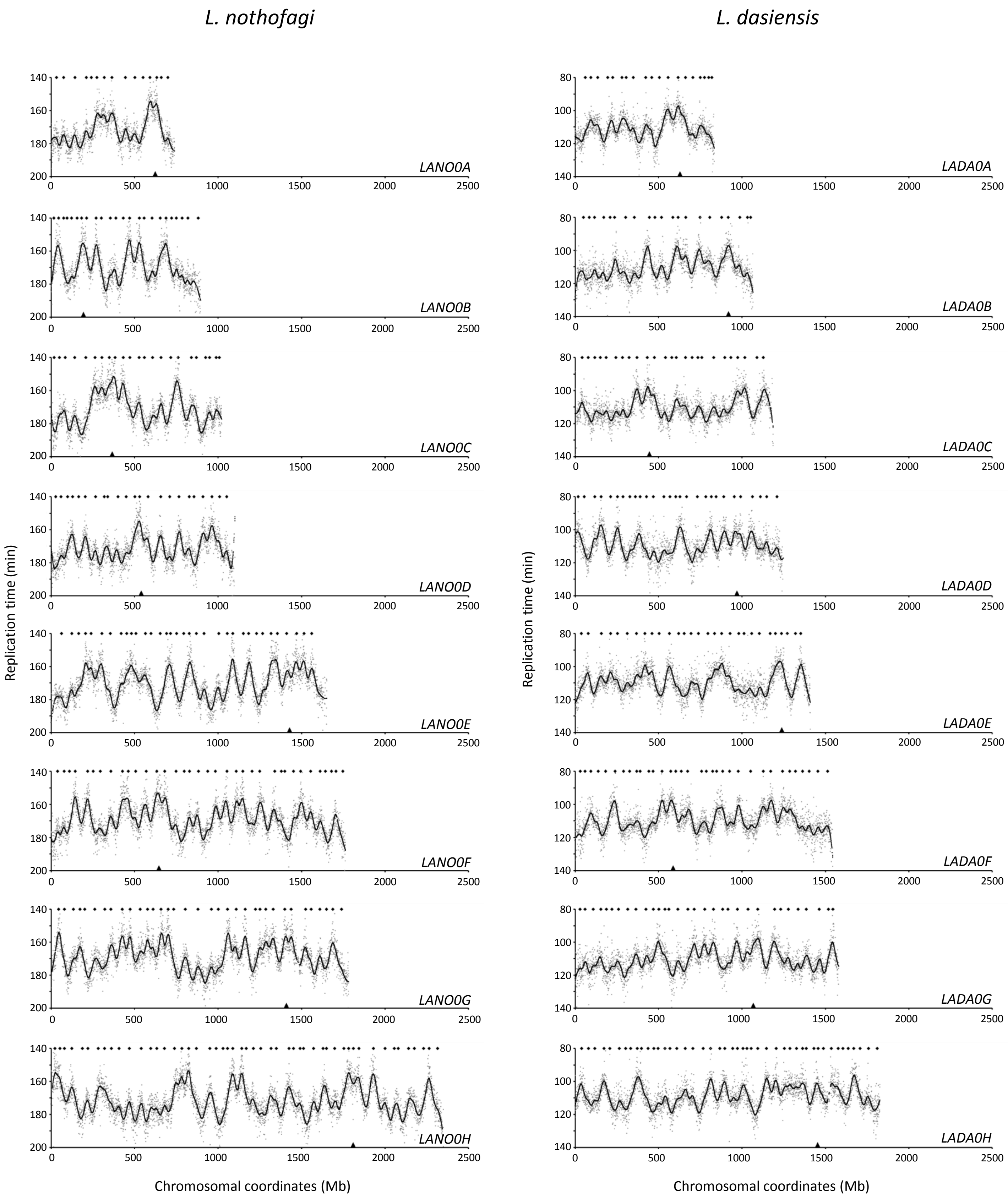
L. nothofagi
L. dasiensis
LANO0A
LANO0B
LANO0C
LANO0D
LANO0E
LANO0F
LANO0G
LANO0H
LADA0A
LADA0B
LADA0C
LADA0D
LADA0E
LADA0F
LADA0G
LADA0H
Replication time (min)
Chromosomal coordinates (Mb)

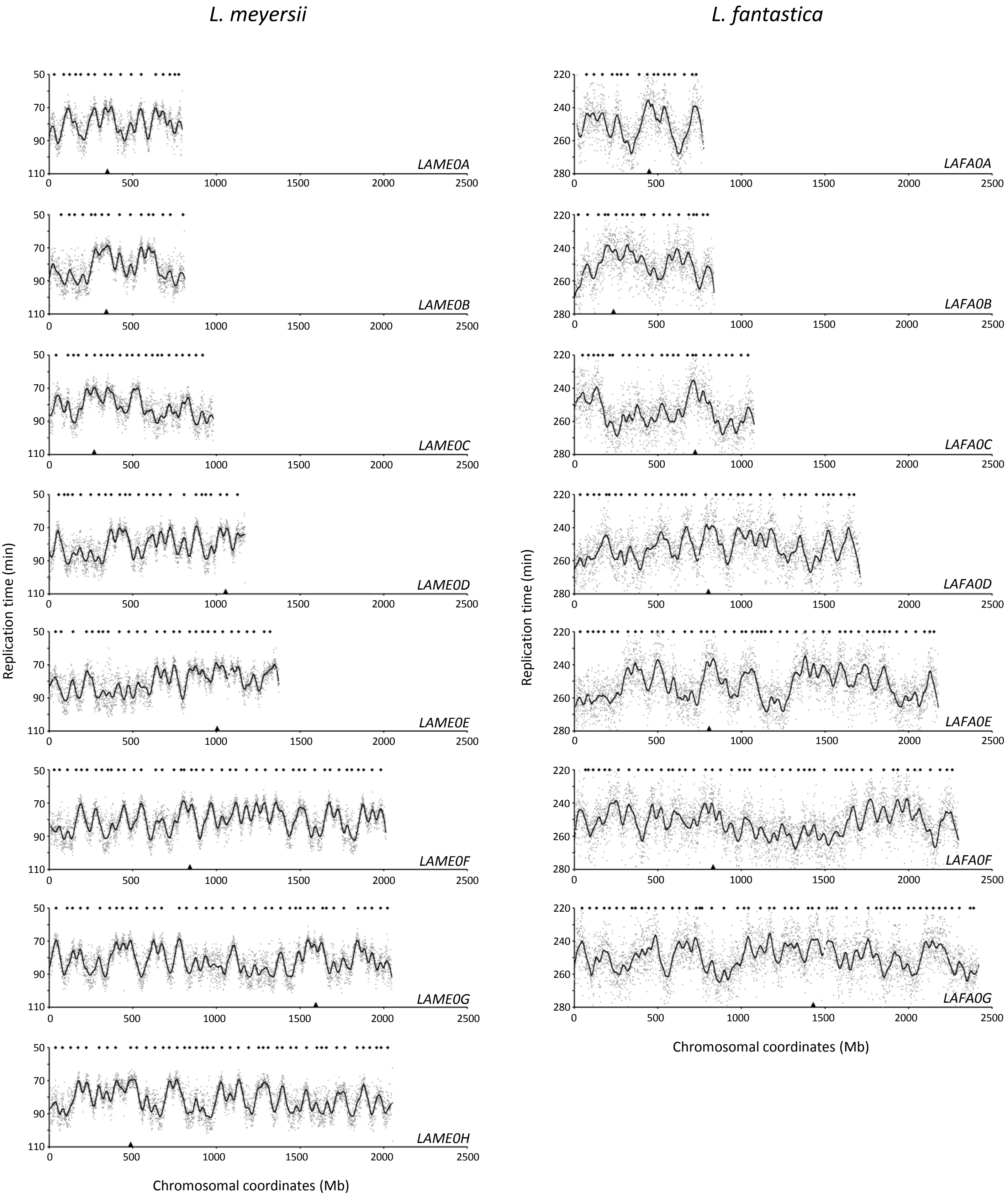

L. meyersii
L. fantastica
LAME0A
LAME0B
LAME0C
LAME0D
LAME0E
LAME0F
LAME0G
LAME0H
LAFA0A
LAFA0B
LAFA0C
LAFA0D
LAFA0E
LAFA0F
LAFA0G
Replication time (min)
Chromosomal coordinates (Mb)

Supplementary Fig. 1: Replication timing programs in 10 *Lachancea* species. Each plot corresponds to a single chromosome. The *x-axis* represents the chromosomal coordinates in kb and the *y-axis* shows the time in minutes from the release of G1 cells in YPD. Black triangles show the position of the centromere. Grey dots correspond to the Trep calculated in 500 bp non-overlaping windows. The black curve indicates the mean replication times obtained by smoothing the Trep data with a loess regression. Black diamonds on the top of each plot indicate the position of the replication origins.

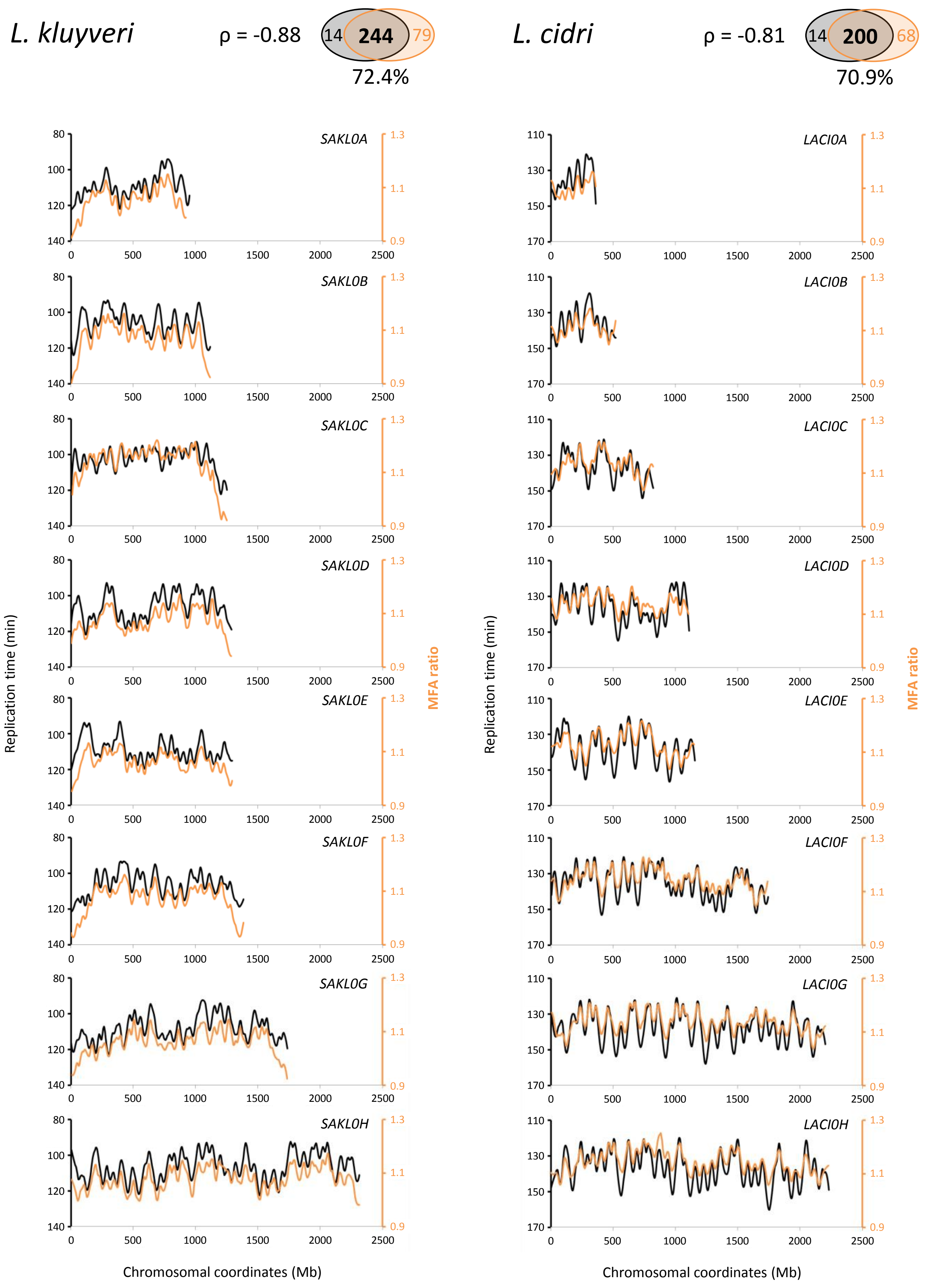
L. kluyveri
ρ = -0.88
14
244
79
72.4%
L. cidri
ρ = -0.81
14
200
68
70.9%
SAKL0A
SAKL0B
SAKL0C
SAKL0D
SAKL0E
SAKL0F
SAKL0G
SAKL0H
LACI0A
LACI0B
LACI0C
LACI0D
LACI0E
LACI0F
LACI0G
LACI0H
Replication time (min)
MFA ratio
Chromosomal coordinates (Mb)

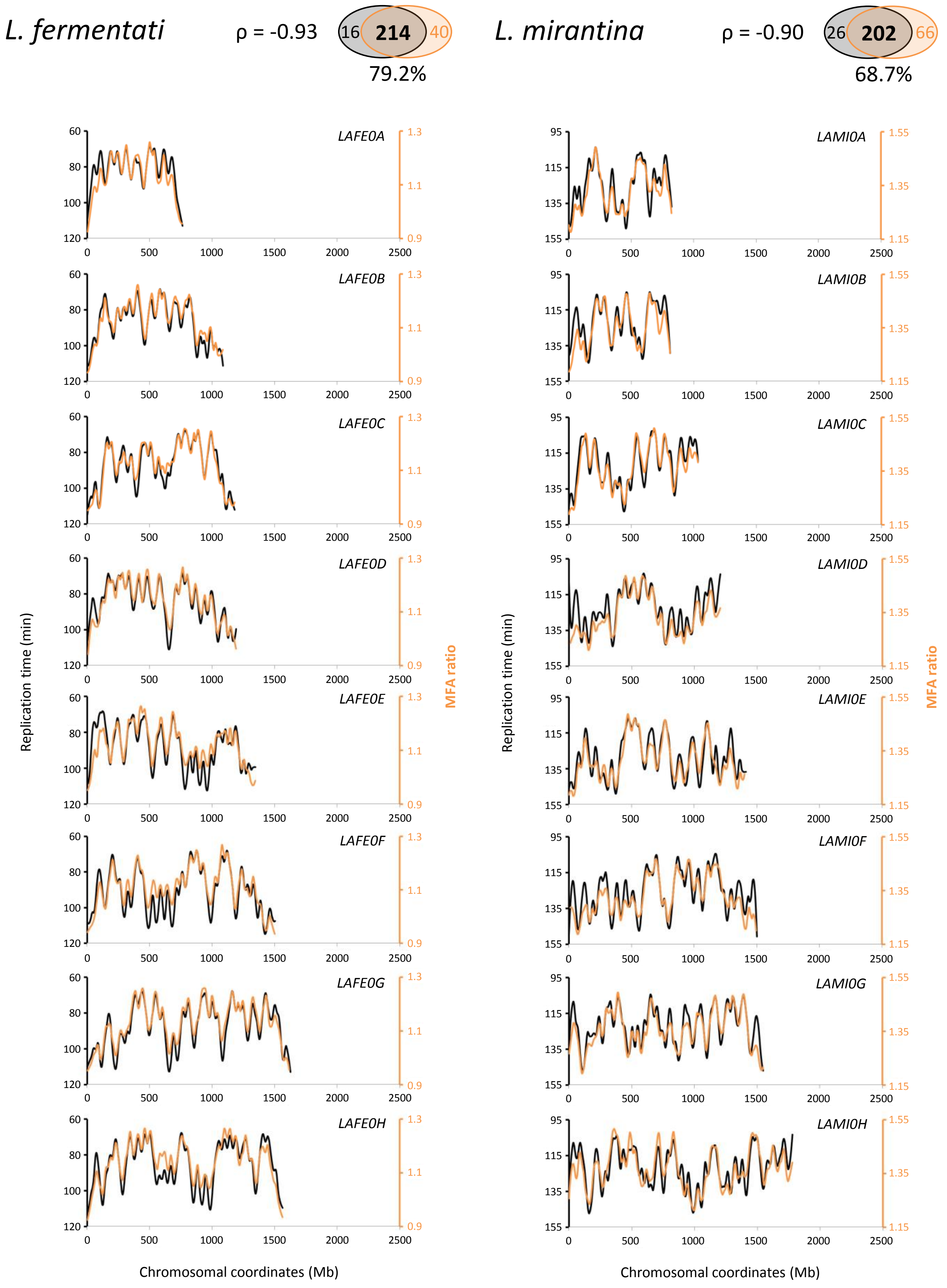

L. fermentati
ρ = -0.93
16
214
40
79.2%
L. mirantina
ρ = -0.90
26
202
66
68.7%
LAFE0A
LAFE0B
LAFE0C
LAFE0D
LAFE0E
LAFE0F
LAFE0G
LAFE0H
LAMI0A
LAMI0B
LAMI0C
LAMI0D
LAMI0E
LAMI0F
LAMI0G
LAMI0H
Replication time (min)
MFA ratio
Chromosomal coordinates (Mb)

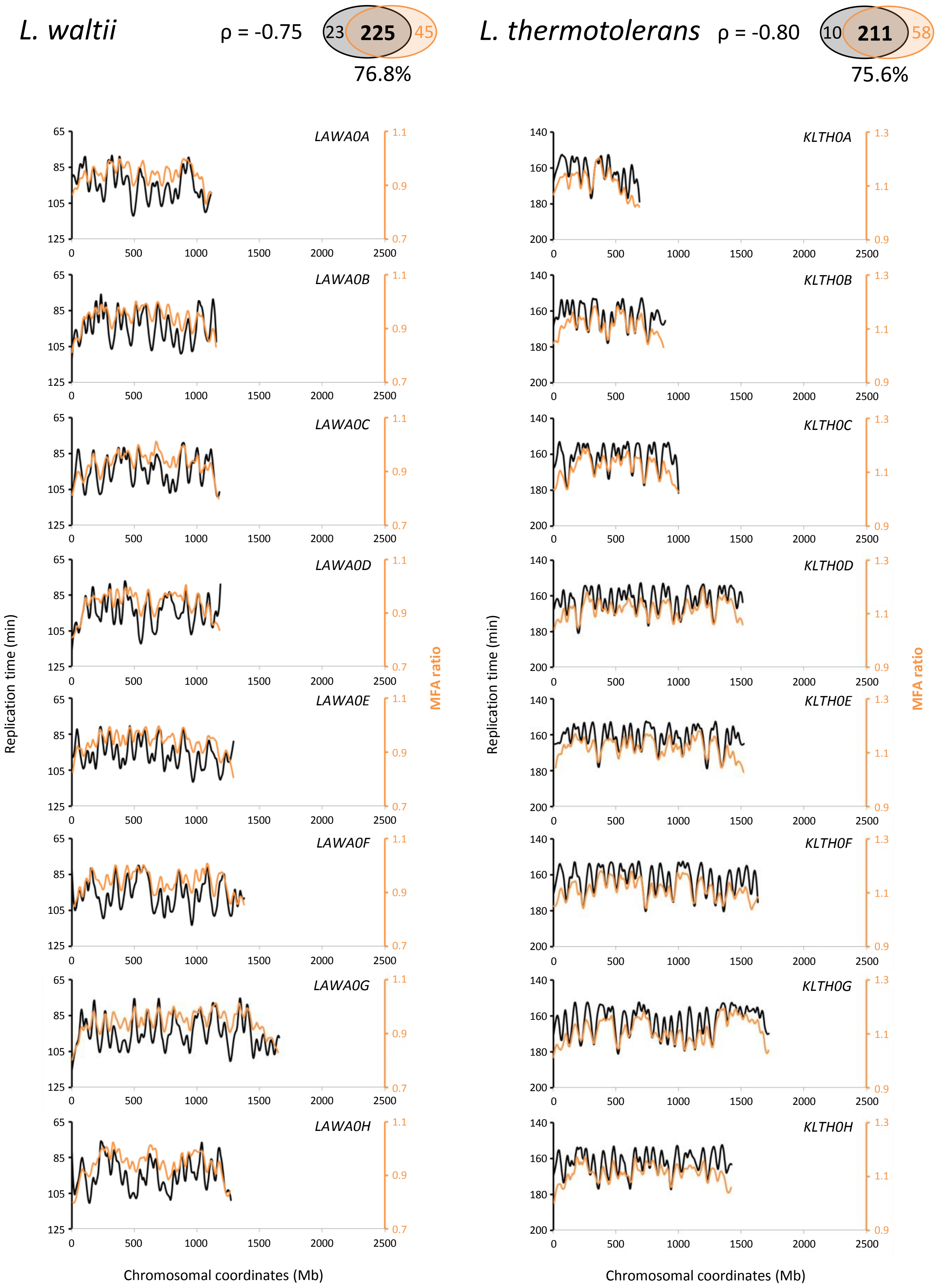

L. waltii
ρ = -0.75
23
225
45
76.8%
L. thermotolerans
ρ = -0.80
10
211
58
75.6%
LAWA0A
LAWA0B
LAWA0C
LAWA0D
LAWA0E
LAWA0F
LAWA0G
LAWA0H
KLTH0A
KLTH0B
KLTH0C
KLTH0D
KLTH0E
KLTH0F
KLTH0G
KLTH0H
Replication time (min)
MFA ratio
Chromosomal coordinates (Mb)
0
500
1000
1500
2000
2500
65
85
105
125
140
160
180
200
1.1
0.9
0.7
1.3
1.1
0.9

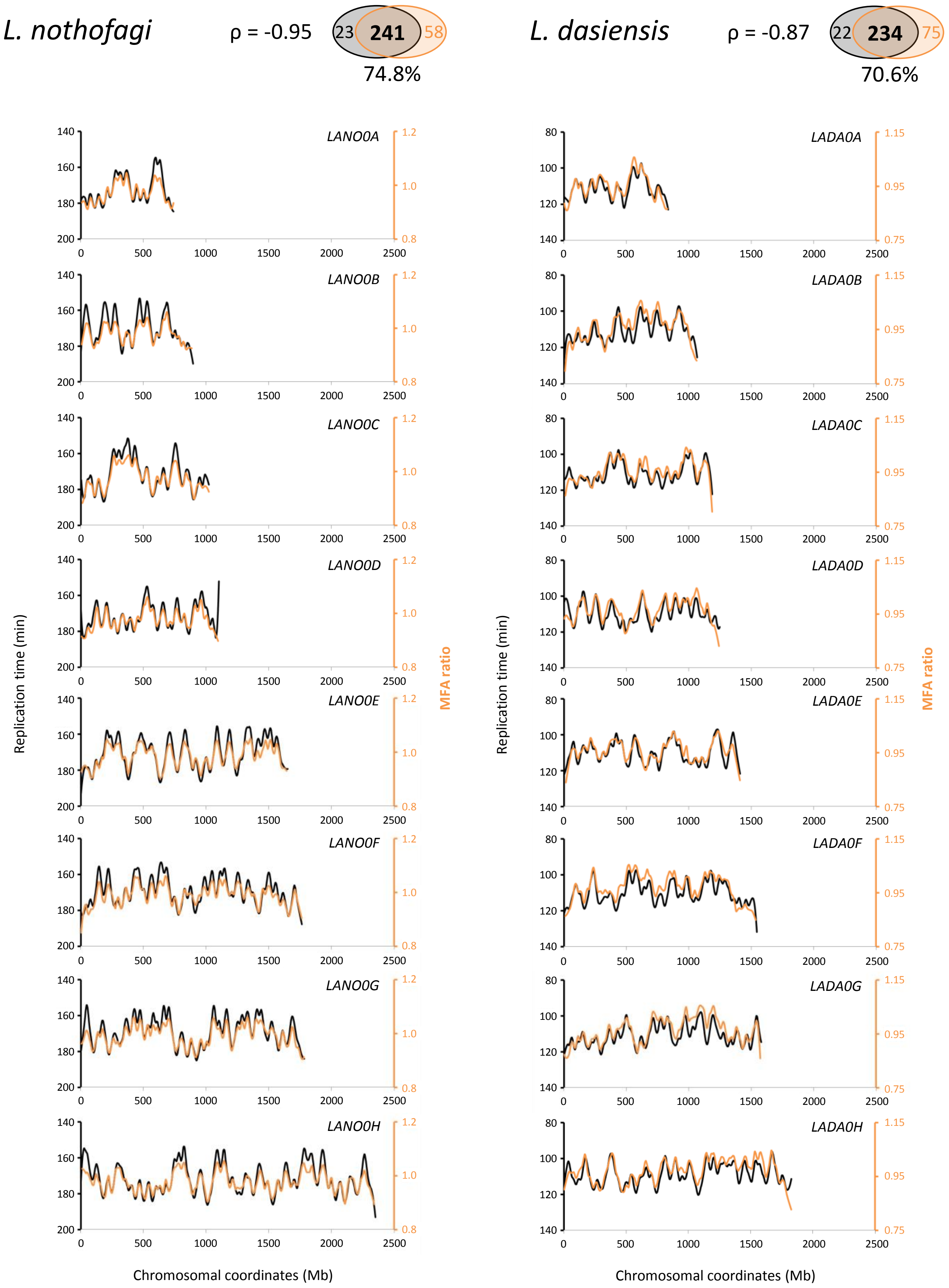
L. nothofagi
ρ = -0.95
23
241
58
74.8%
L. dasiensis
ρ = -0.87
22
234
75
70.6%
LANO0A
LANO0B
LANO0C
LANO0D
LANO0E
LANO0F
LANO0G
LANO0H
LADA0A
LADA0B
LADA0C
LADA0D
LADA0E
LADA0F
LADA0G
LADA0H
Replication time (min)
MFA ratio
Chromosomal coordinates (Mb)

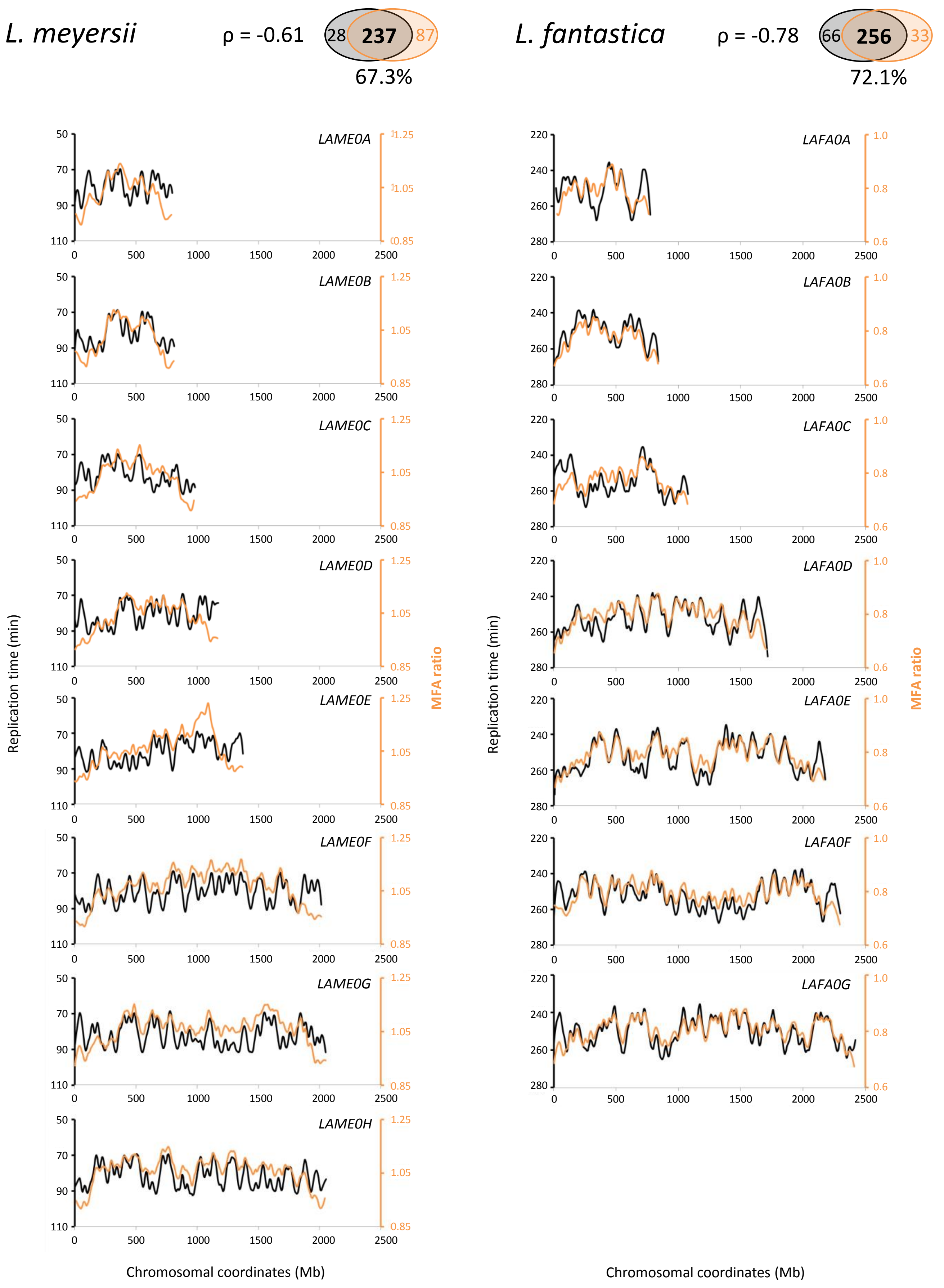
L. meyersii
ρ = -0.61
28
237
87
67.3%
L. fantastica
ρ = -0.78
66
256
33
72.1%
LAME0A
LAME0B
LAME0C
LAME0D
LAME0E
LAME0F
LAME0G
LAME0H
LAFA0A
LAFA0B
LAFA0C
LAFA0D
LAFA0E
LAFA0F
LAFA0G
Replication time (min)
MFA ratio
Chromosomal coordinates (Mb)
0
500
1000
1500
2000
2500
50
70
90
110
1.25
1.05
0.85
220
240
260
280
1.0
0.8
0.6

Supplementary Fig. 2: Overlay of the mean replication time and the MFA profile in all *Lachancea* species. Each plot shows the mean replication time (black) and MFA profile (orange) for a single chromosome whose name is indicated in the top right of each graph. The Spearman correlation coefficient values (Rho) correspond to the genome-wide comparison of the two profiles. The Venn diagrams give the overlap in absolute number and percentage between the two sets of origins identified by each method.

a

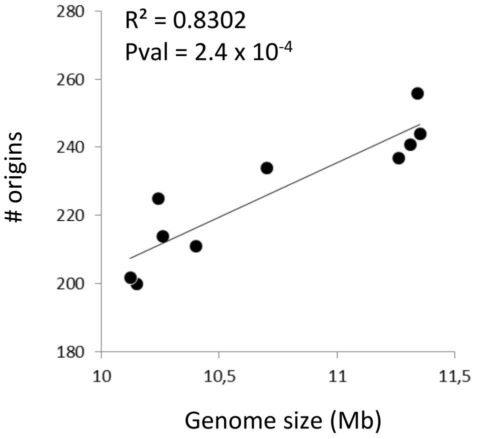

b

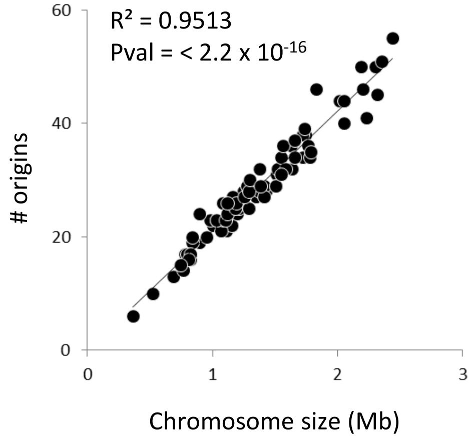

c

Species	R^2	Pval
LAKL	0.96	2.9E-05
LACI	0.98	2.1E-06
LAFE	0.94	5.3E-05
LAMI	0.97	5.2E-06
LAWA	0.86	8.2E-04
LATH	0.98	2.7E-06
LANO	0.96	1.9E-05
LADA	0.91	2.3E-04
LAME	0.98	3.3E-06
LAFA	1.00	5.8E-07
All	0.95	< 2.2E-16

d

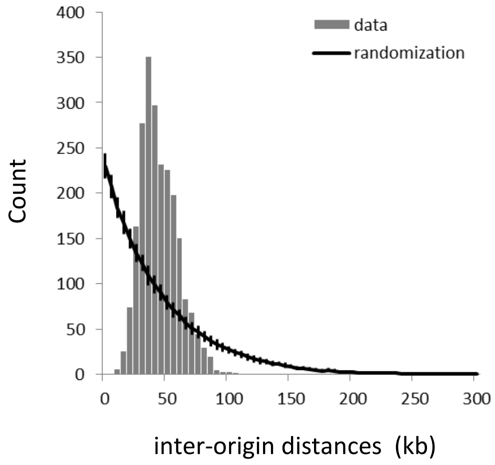

Supplementary Fig. 3: Distribution of origins along chromosomes. **a.** Correlation between the number of active replication origins and the genome size. Each dot corresponds to a species. The Pearson correlation coefficient and its associated P-value are indicated. **b.** Correlation between the number of active replication origins and the chromosome size. Each dot corresponds to a single chromosome. The Pearson correlation coefficient and its associated P-value are indicated. **c.** Pearson correlation coefficients and associated *P* values between the number of active replication origins and the chromosome size for each species. **d.** Comparison between the observed distribution of inter-origin distances (grey histogram) and expected distances if origins were randomly localized (black curve) in the 10 *Lachancea* species. To generate the black curve, origin positions were randomized 100 times in each of the ten *Lachancea* genomes. A similar distribution is also observed for each of the ten species taken individually.

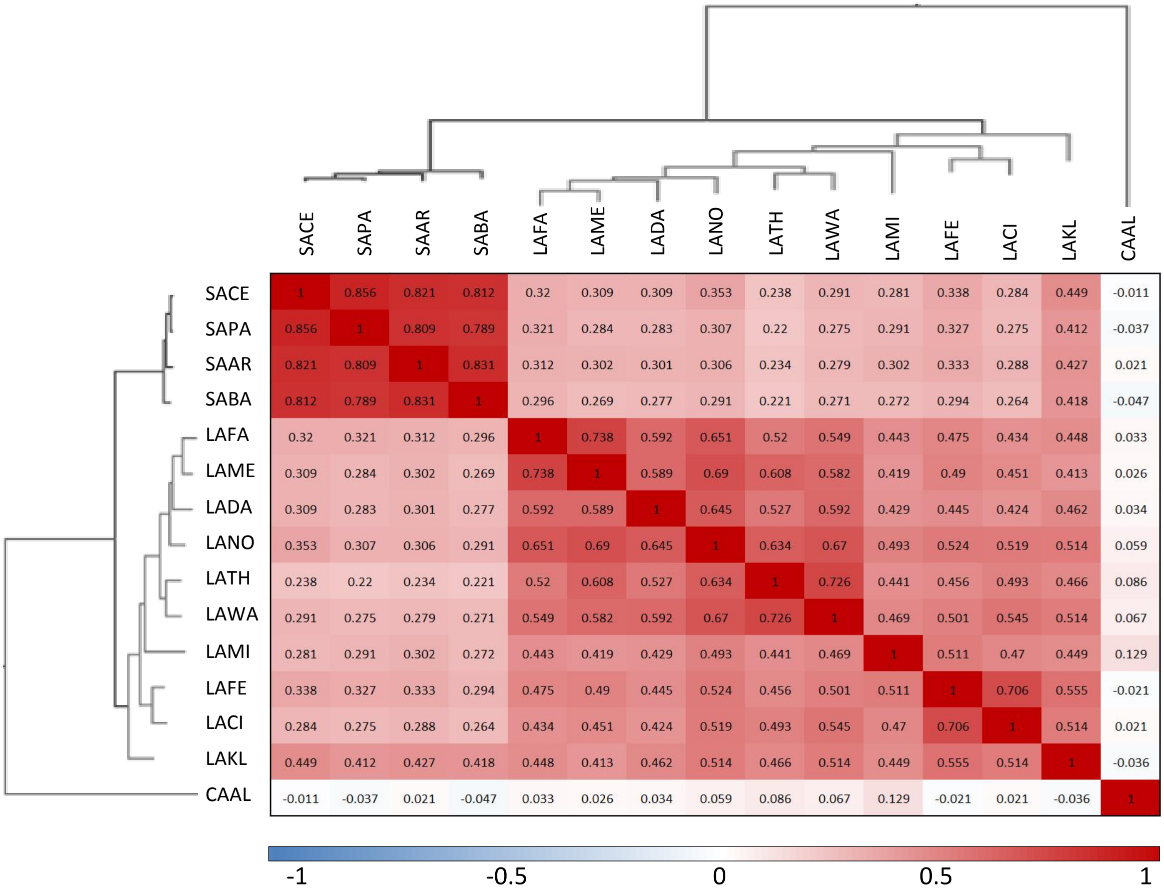

Supplementary Fig. 4: Matrix of pairwise Spearman's rank correlation coefficients between replication timing programs in Saccharomycotina species. The species tree is taken from Vakirlis *et al.,* Genome Res, 2016.

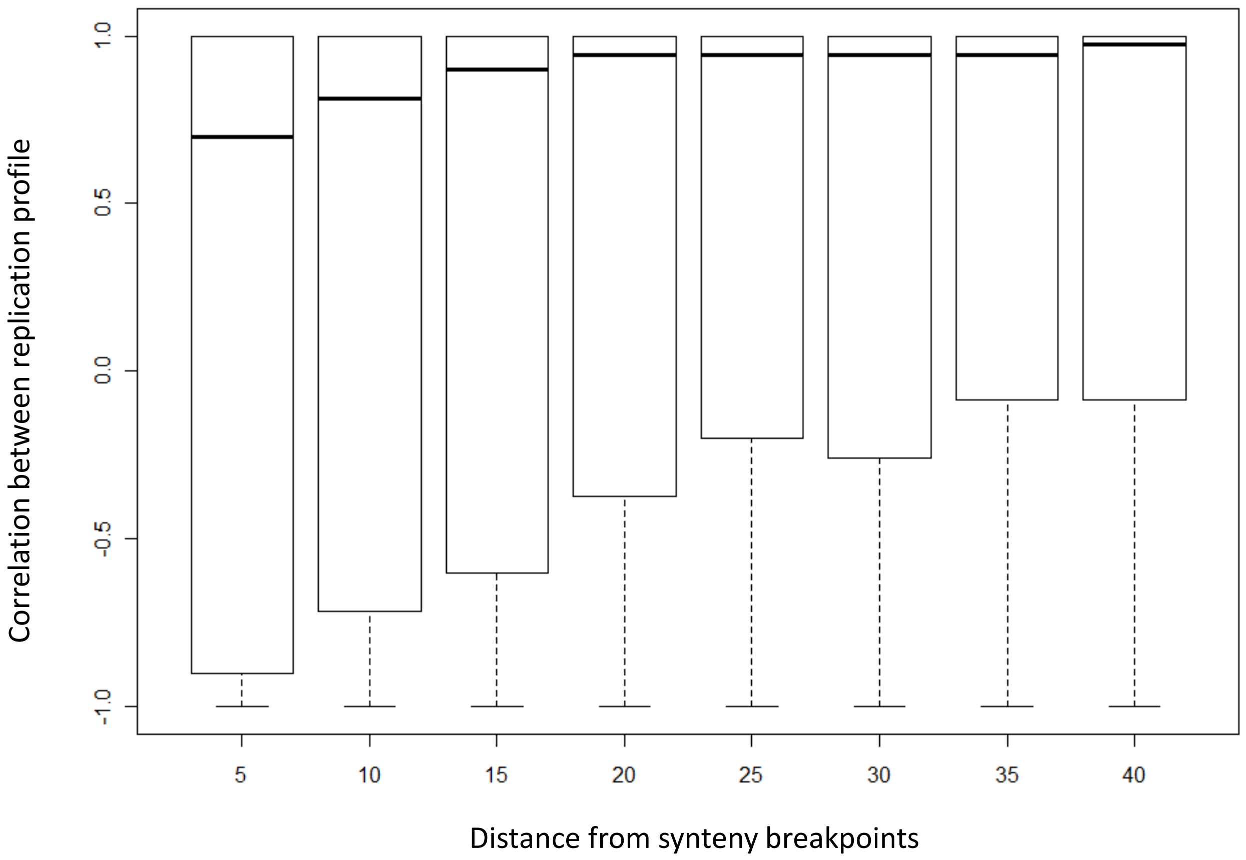

Supplementary Fig. 5: Correlation levels between replication profiles at increasing distance from synteny breakpoints. All pairwise comparisons between the 10 *Lachancea* species are merged together. Each boxplot corresponds to a 5 gene non-overlapping sliding window. Whiskers are defined by selecting the value within the range of the 75th and 25th percentile plus or minus 1.5 x (75th percentile – 25th percentile) respectively, and at the maximum distance from the median.

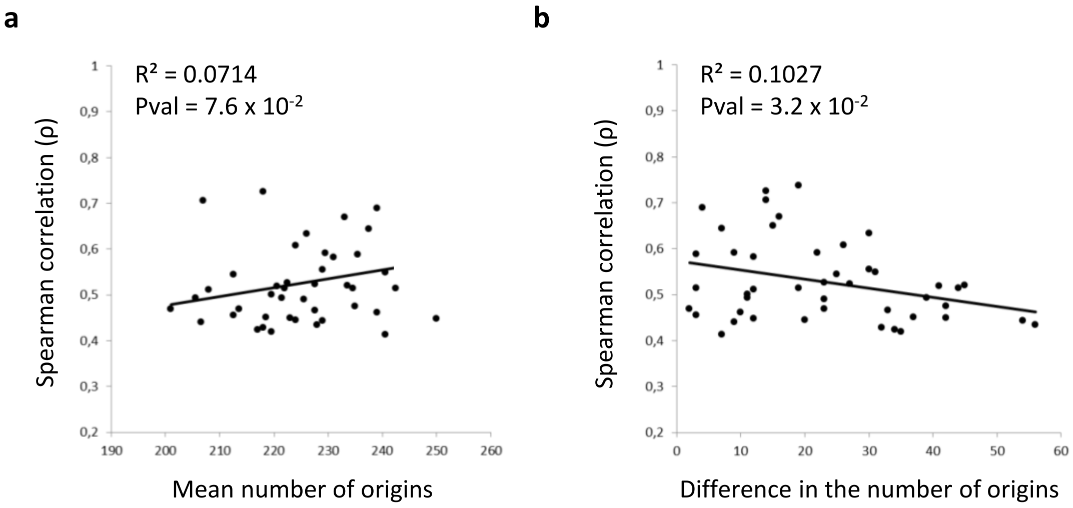

Supplementary Fig. 6: Correlation between pairwise replication correlation coefficients and the number of active origins. Each dot corresponds to a pairwise comparison between two species. **a.** Pairwise rank coefficients as a function of the average origin number between the pair of compared species. **b.** Pairwise rank coefficients as a function of the difference in origin number between pairs of compared species.

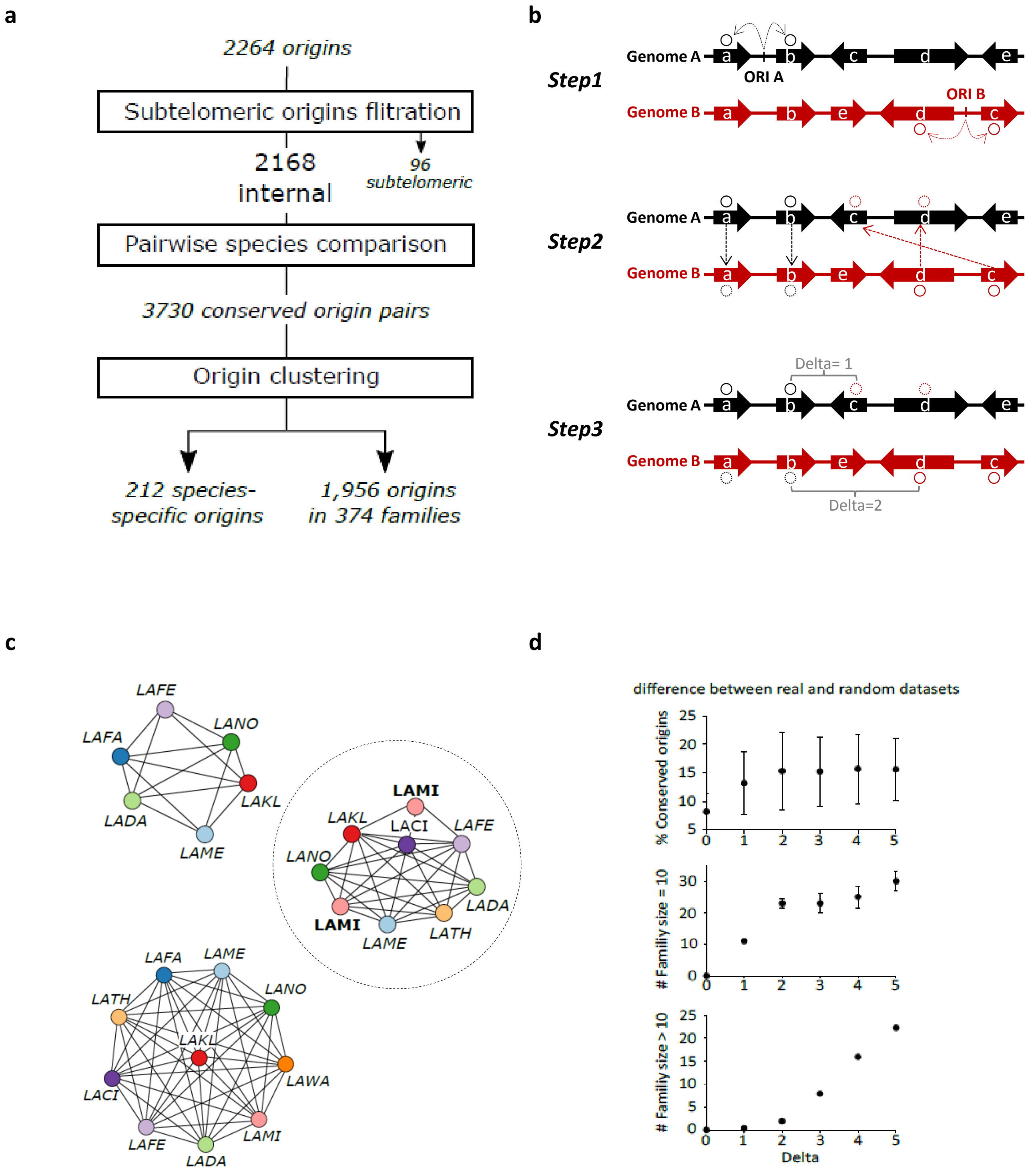
a
2264 origins
Subtelomeric origins flitration
2168
internal
96
subtelomeric
Pairwise species comparison
3730 conserved origin pairs
Origin clustering
212 species-specific origins
1,956 origins in 374 families
b
Step1
Genome A
ORI A
Genome B
ORI B
Step2
Step3
Delta= 1
Delta=2
c
LAFE
LANO
LAFA
LAKL
LADA
LAME
LAMI
LACI
LATH
LAWA
d
difference between real and random datasets
% Conserved origins
# Familiy size = 10
# Familiy size > 10
Delta

Supplementary Fig. 7: Families of orthologous replication origins. **a.** Flowchart representing the different steps in family construction. We excluded 96 subtelomeric origins due to poor synteny conservation and clustered the remaining 2,168 internal origins, representing 96% of the total, into 374 multi-origin families comprising 1,956 origins (90%) and 212 species-specific singleton origins (10%). **b.** Definition of a pair of orthologous origins. Genes, named a to e, are represented by plain arrows . Genome A and B differ by an inversion of genes c, d and e. Step1: The resident replication origins (ORI A and ORI B, symbolized by open circles) are anchored to their neighboring genes, both on genome A and B. Step 2: Anchored origins from genome A are projected in the syntenic homologs in genome B, and reciprocally. Projected origins are represented by dashed circles. Step 3: The distance in number of genes (Delta) between anchored and projected origins is calculated in both genomes. The two resident origins are considered as orthologous if both Deltas are ≤ 2. **c.** Examples of 3 origin families represented as connected components. Species names are abbreviated as in Fig. 3. The circled family contains two origins from the species LAMI resulting from the presence of two closely located origins assembled into a single connected component during the aggregation of orthologous origin pairs. Similar cases are found in 27 out of the 374 families. **d.** Estimation of the optimal number of syntenic genes between 2 origins allowed for origin clustering. The threshold of delta=2 syntenic genes is the smallest value that maximized the median difference in the percentage of conserved origins between the real and 100 random datasets (top), maximized the median difference of the number of families comprising 10 origins between real and a 100 random datasets (middle) and minimized the number of families with more than 10 members in the real dataset (bottom). For the two upper plots, the random datasets correspond to simulations based on a null model that preserves the same distribution of inter-origin distances as in real genomes; error bars represent the standard deviation of the differences.

a **b**

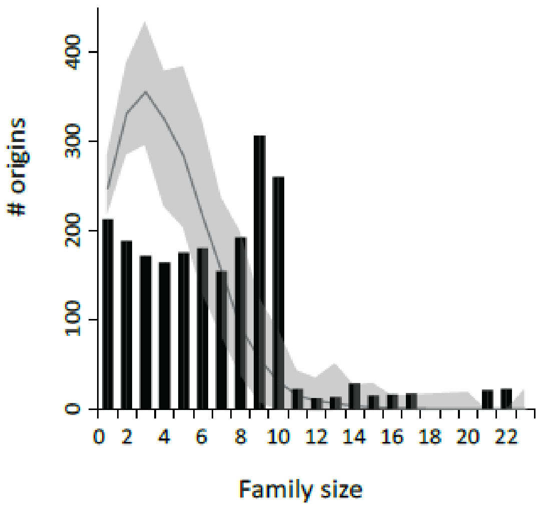

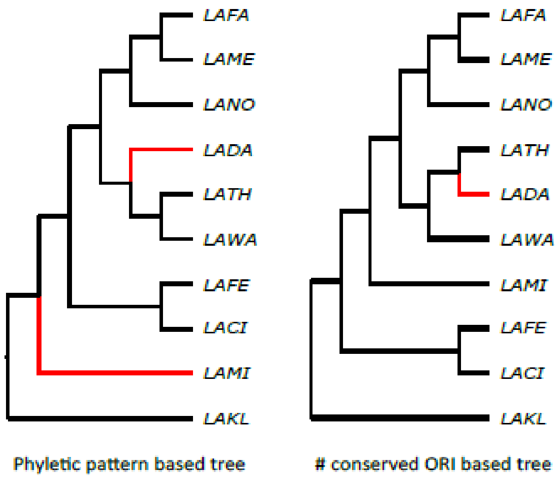

Supplementary Fig. 8: Characterization of the families of orthologous replication origins. **a.** Distribution of the number of origins per family. The black histogram represents the distribution of the actual family sizes while the curve represents the distribution of the median and standard deviation (grey area) obtained from 100 simulations based on a null model with the same distribution of inter-origin distances as in real genomes. **b.** Neighbor Joining phylogenetic trees based on the composition of orthologous origin families based on the phyletic patterns of origin families (left) and on a distance matrix representing the proportion of conserved origins between pairs of species. The two resulting tree topologies are very similar to the topology of the reference species tree based on the concatenation of 3,598 orthologous protein sequences (Vakirlis *et al.,* Genome Res, 2016). The only few bipartitions being different from the species tree are highlighted in red. Species names are abbreviated as in Fig 3.

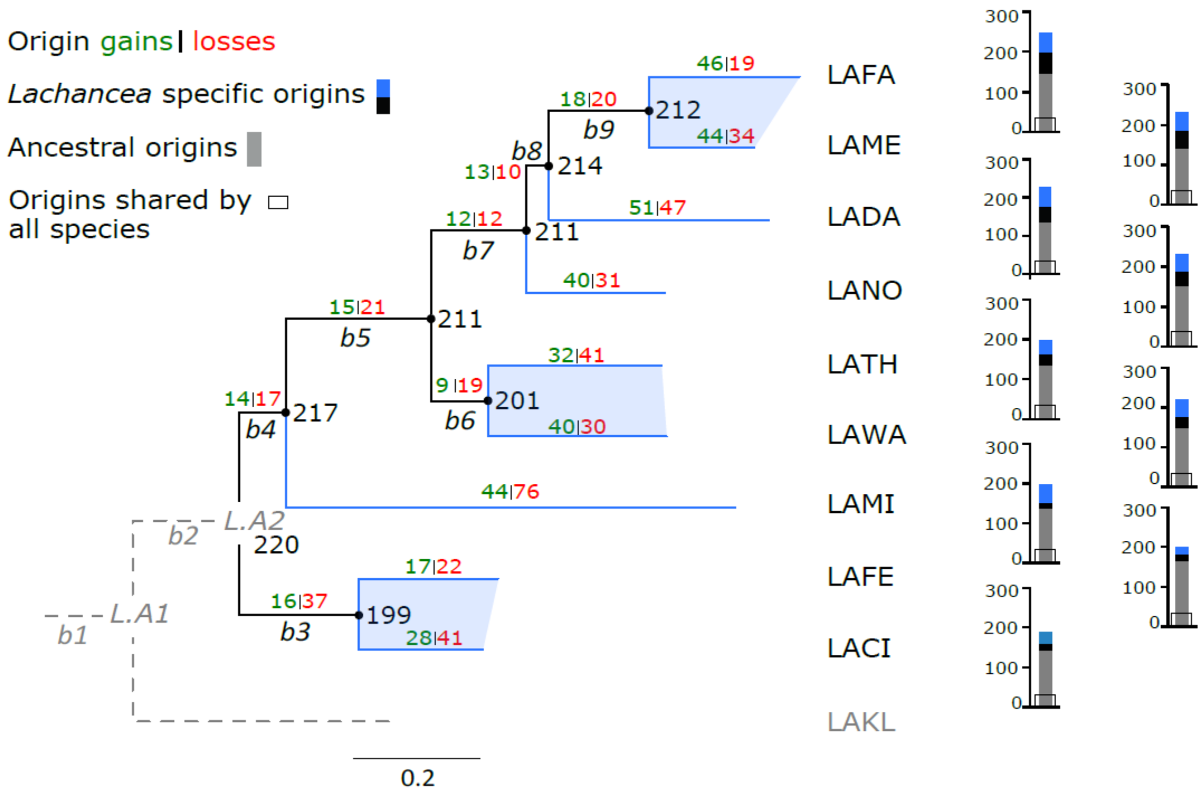

Supplementary Fig. 9: Individual contribution of *Lachancea* species to the evolutionary dynamics of replication origins. The phylogenetic tree on the left is taken from Fig. 3a. Histograms on the right indicate the number of active origins that were vertically inherited from the *L.A2* ancestor (in grey), gained on internal branches of the tree (in black) and gained on terminal branches (in blue). The proportion of ancestral origins retained in all nine genomes (not considering LAKL) is indicated by the open frame.

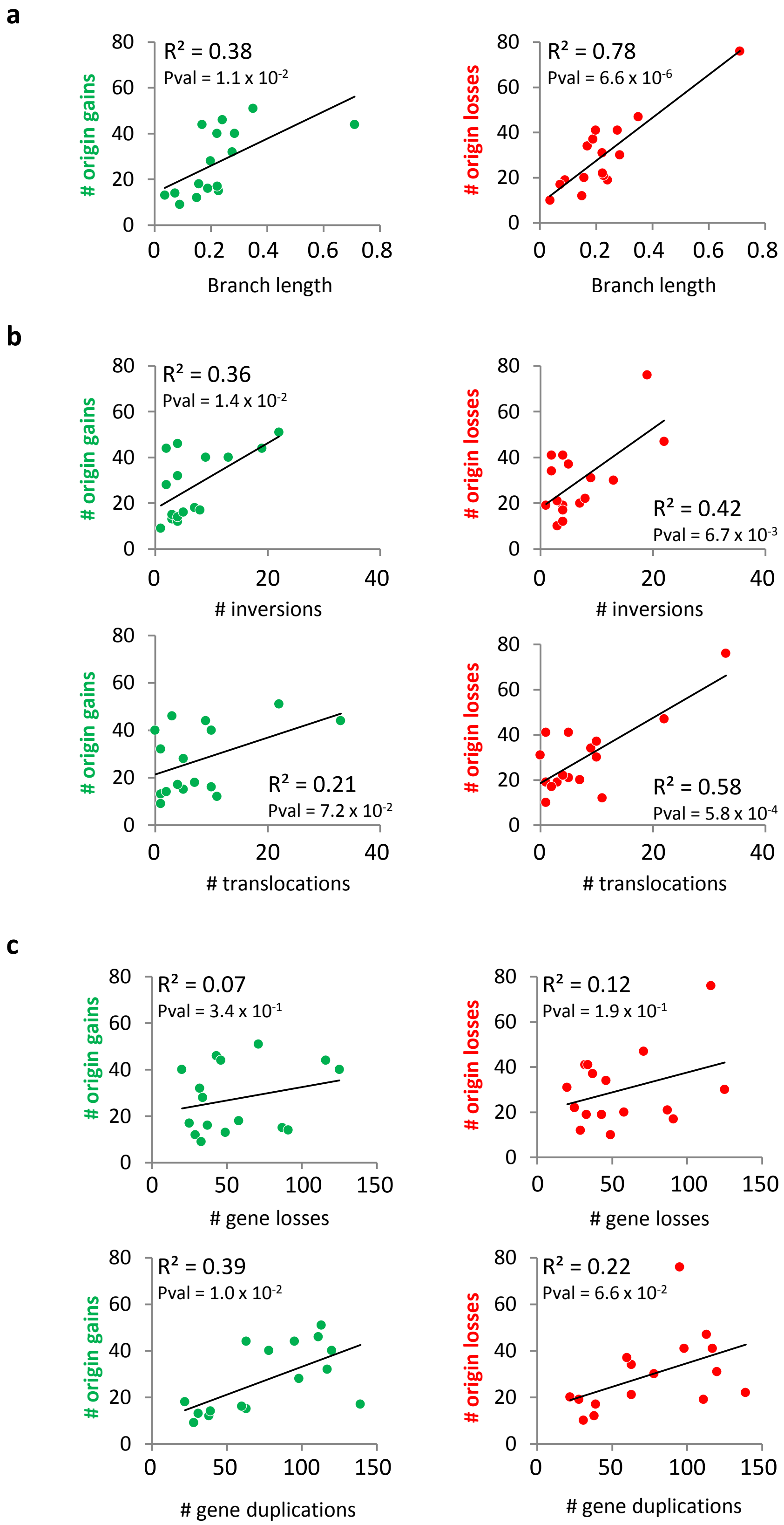

Supplementary Fig. 10: Relationship between origin gains and losses and mutational events. Correlations between both gains and losses and **a.** non-synonymous substitution rates, **b.** balanced chromosomal rearrangements and **c.** gene losses and gene duplications. Pearson correlation coefficients and associated P-values are indicated.

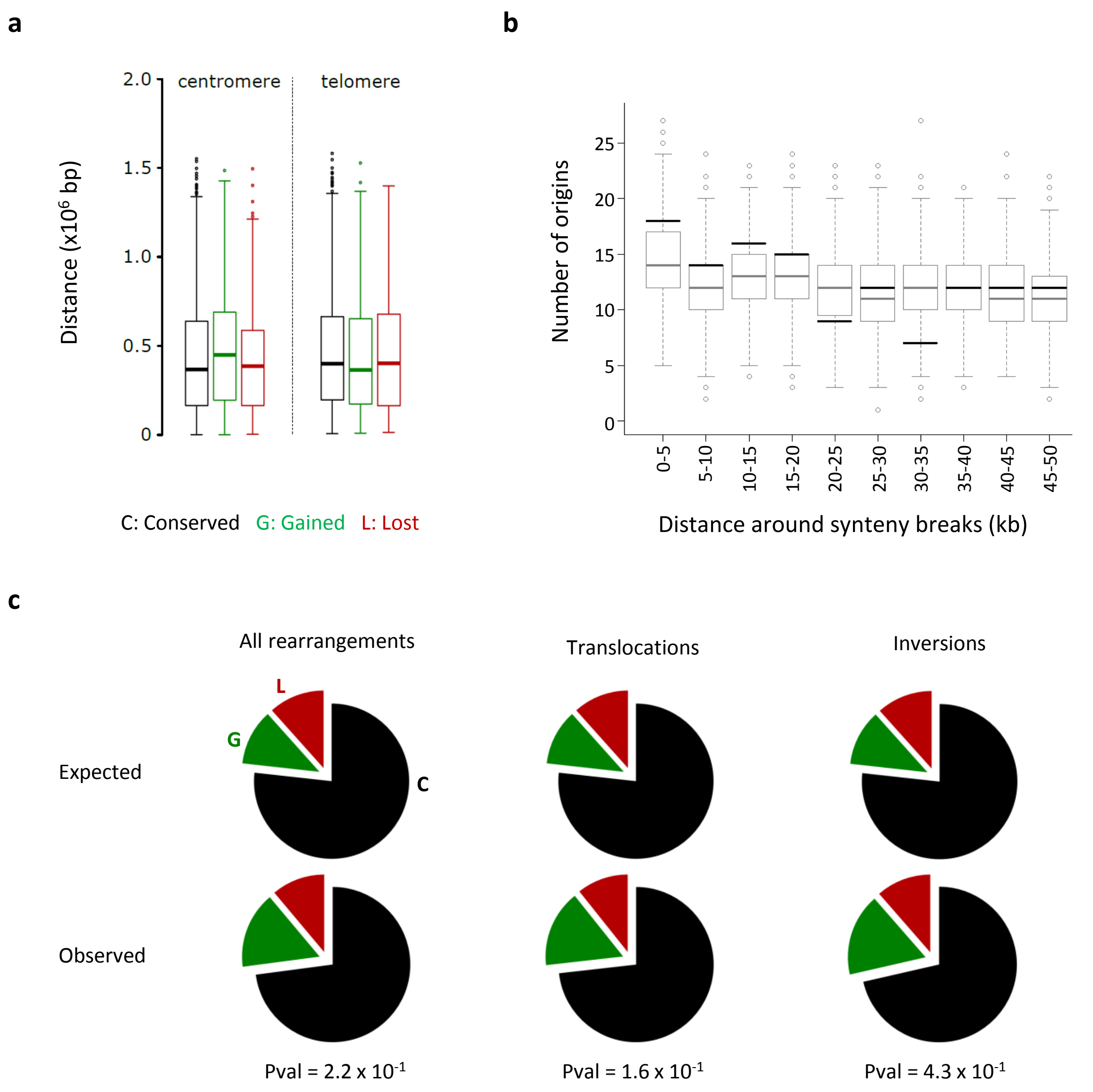

Supplementary Fig. 11: Distances between active replication origins and specific chromosomal sites. We focus here on the most recent events that occurred on the six terminal branches of the tree that lead to the three most closely related pairs of species (see blue shaded area in Fig. 3). **a.** Boxplots showing the distance distribution between conserved (C), gained (G) and lost (L) origins and both their respective centromere (left panel) and closest telomere (right panel). **b.** Number of origins found in non-overlapping 5 kb windows centered around synteny breakpoints. The observed data are indicted by the black dashes and the boxplots show the distribution from 1000 simulations based on the null model that preserves the same distribution of inter-origin distances as in real genomes. For a and b, Whiskers are defined by selecting the value within the range of the 75th and 25th percentile plus or minus 1.5 x (75th percentile – 25th percentile) respectively, and at the maximum distance from the median. **c.** Comparisons between the expected and observed numbers of C, G and L origins in 50 kb windows centered around the rearrangement breakpoints for the three most closely related pairs of species. P-values were calculated from Goodness of Fit Chi-Square tests. The theoretical values correspond to the number of different types of origins expected at random in the same total window size than for the observed values. The same calculations were also performed in each individual species and no significant difference was found between observed and expected number of origins.

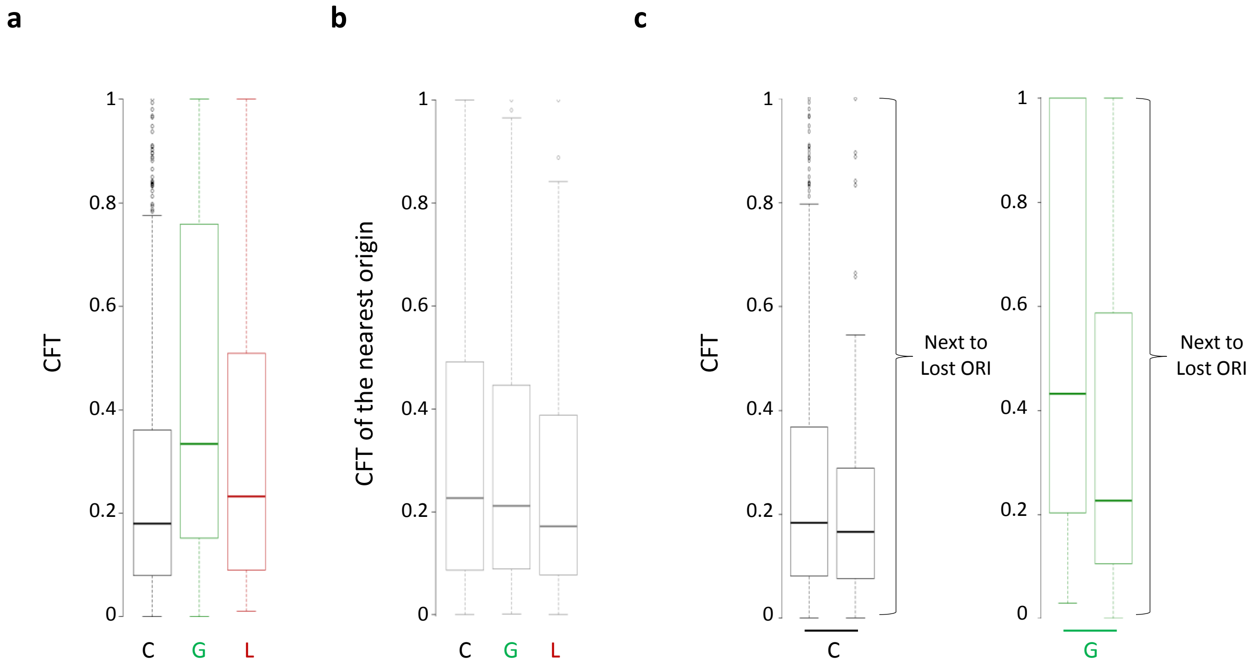

Supplementary Fig. 12: Characteristic firing times (CFT) of conserved and recently gained and lost origins. We focus here on the most recent events that occurred on the six terminal branches of the tree that lead to the three most closely related pairs of species (see blue shaded area in Fig. 3). **a.** Distribution of the CFT of conserved (C), gained (G) and lost (L) origins. **b.** CFT distributions of the neighboring origins flanking C, G and L origins. **c.** Split distributions of the CFT of C and G origins based on the category of their neighboring origins (ORI). Boxplot whiskers are defined by selecting the value within the range of the 75th and 25th percentile plus or minus 1.5 x (75th percentile – 25th percentile) respectively, and at the maximum distance from the median.

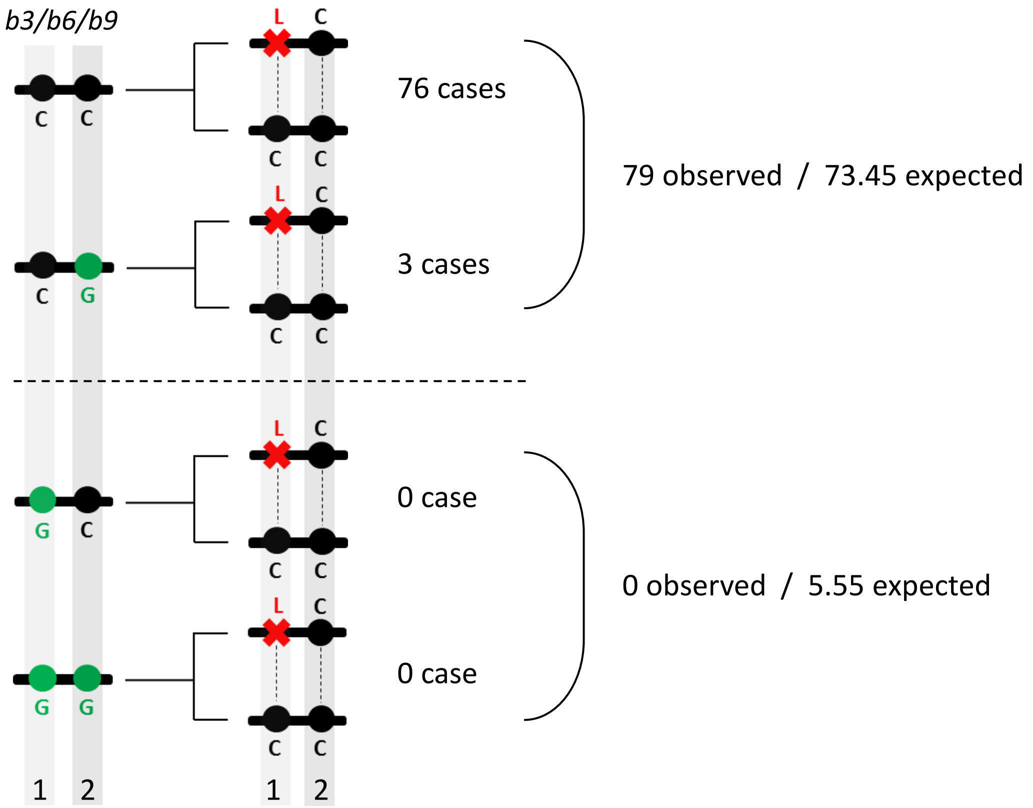

Supplementary Fig. 13: Categories of origins that were lost in the terminal branches leading to the 3 pairs of sister species *L. fantastica,/L. meyersii, L. thermotolerans/L.* waltii and *L. fermentati/L. cidri*. The 79 origins losses (L) were categorized based on the state, conserved (C) or gained (G), of their ancestral origins in the preceding internal branches (*b3, b6* or *b9* in Fig. 3a). Position 1 (light-grey shading) spots the location of the origin losses and position 2 (dark-grey shading) the location of the nearest neighboring origin. The expected proportion of each category was estimated from the proportion of conserved and gained origins in the corresponding ancestral genomes. The difference between the expected and observed numbers of categories is statistically significant (Chi² $P = 0.04927$).

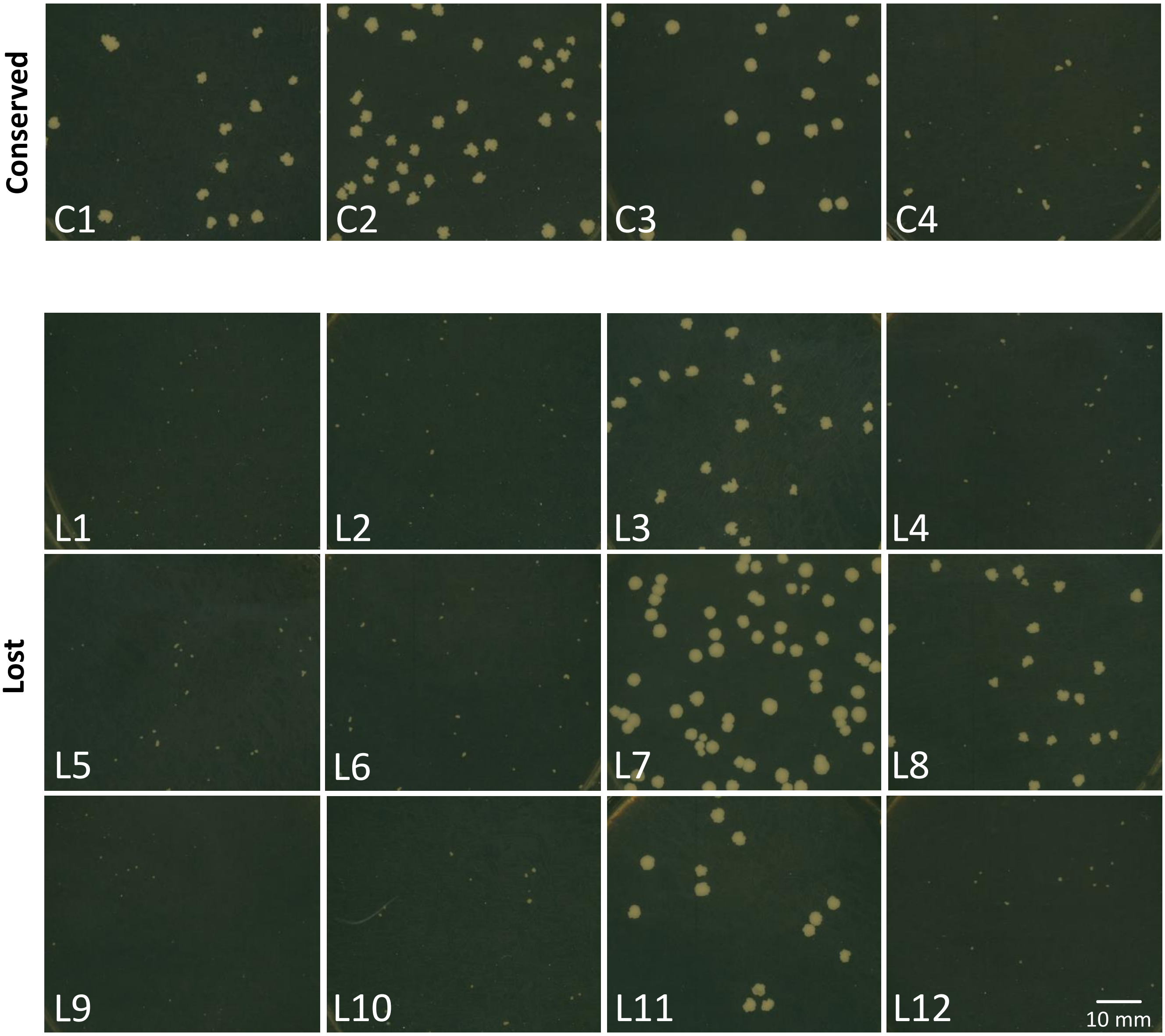

Supplementary Fig. 14: ARS Assay. Snapshot of the plates 4 days after transformation of *L. thermotolerans.* C1 to C4 correspond to cells transformed with centromeric plasmids into which 4 origins conserved between *L. thermotolerans* and *L. waltii* were cloned. L1-L12 correspond to cells transformed with centromeric plasmids into which regions of lost origins in *L. thermotolerans,* but chromosomally active in *L. waltii,* were cloned. Scale bar in L12 is the same for all pictures.

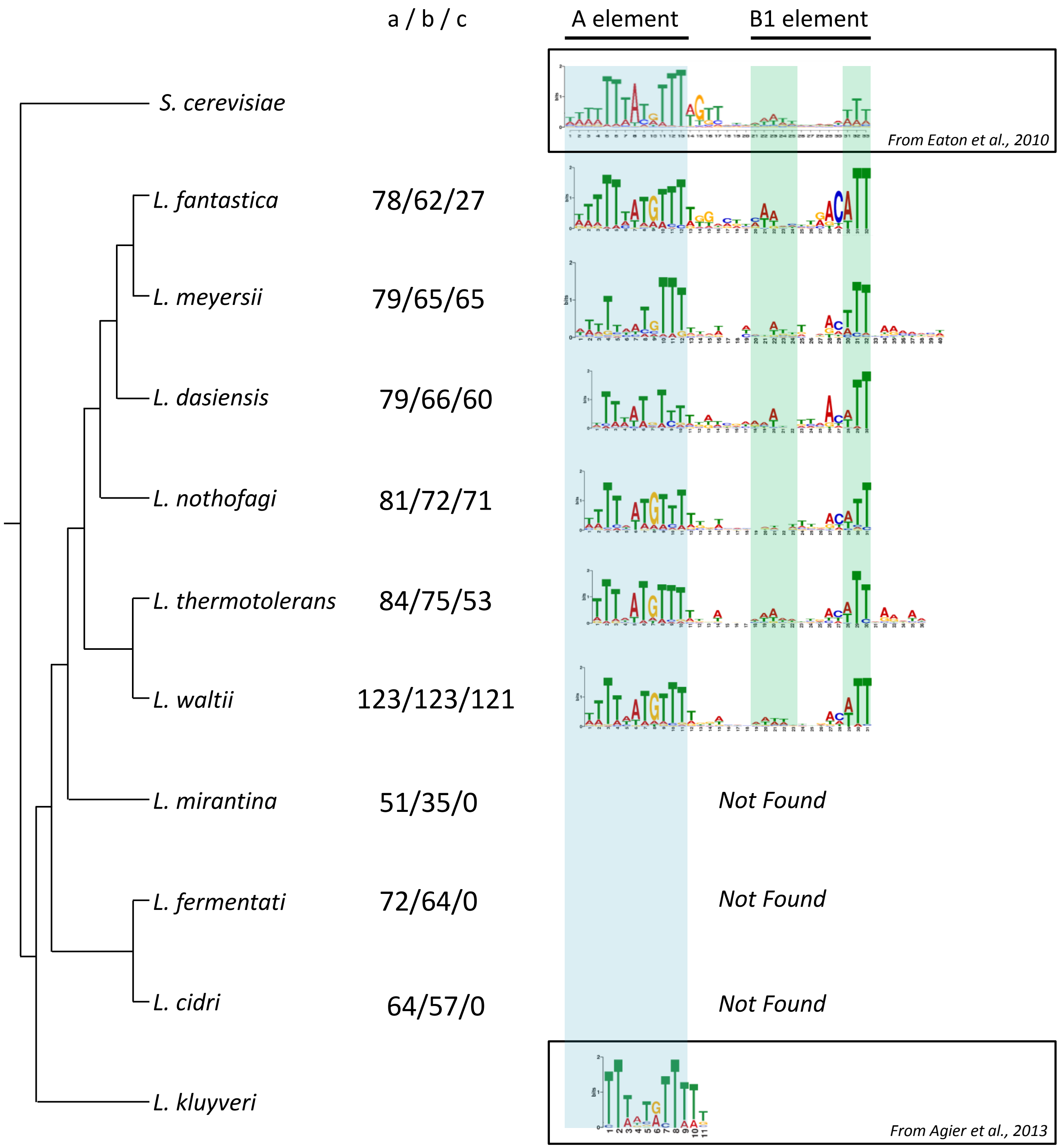

Supplementary Fig. 15: Alignment of ARS consensus sequence (ACS) for the 10 *Lachancea* species and *S. cerevisiae*. The *S. cerevisiae* motif was taken from Eaton *et al.* (Genes Dev, 2010) and the *L. kluyveri* motif was taken from Agier *et al.* (Genome Biol Evol, 2013). The ACS A element (blue box) and the B1 element (green boxes) are represented as illustrated in Di Rienzi *et al.* (Genome Res, 2012). a/b/c: number of origin families with one member having both an ACS in *L. waltii* and an orthologous origin within the alternate species/number of regions conserved in strict synteny between *L. waltii* ACS and the alternate species/number of detected ACS. We found no motif in *L. kluyveri* however, the A element of the ACS previously reported in *L. kluyveri* is similar to the *L. waltii*/*L. thermotolerans*/*S. cerevisiae* motif

a

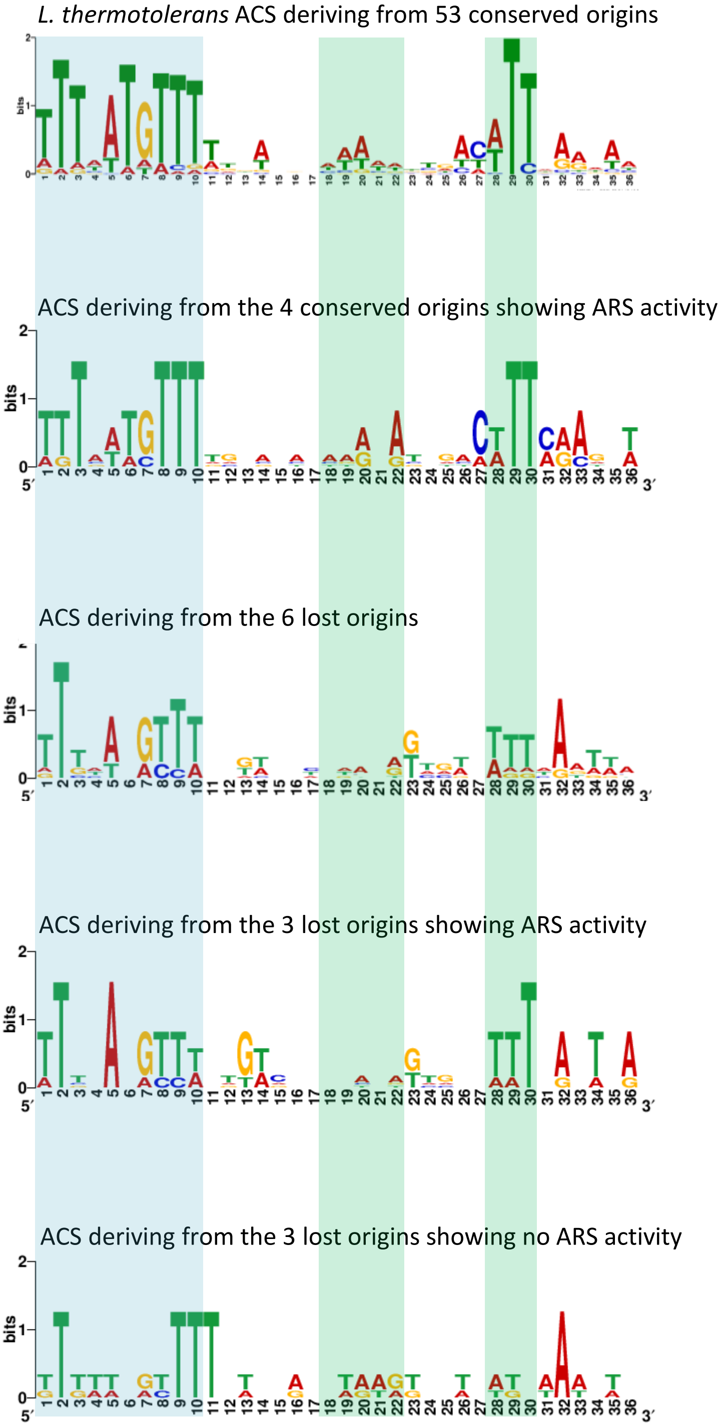

b

```
           1        10        20        30    36
           |--------+---------+---------+-----|
    CONS4  TTTAATGTTTTCCCACAAAAAATTGACATTCGAAAT
    CONS2  TTTAATGTTTAACATTTCGAAAGGGCCTTTCGATCA
  CONS1.1  TTTATAGTTTTGTAGAGTAGTACTGACTTTCAAGAT
    CONS3  TTTCATGTTTGGGGGACATGGATCTACTTTAAAGTT
  CONS1.2  AGTGTTCTTTTGAATATAAATGTCATAATTAACGTA
Consensus  ttT.ttgTTTtg.a.a.aaatatc.actTTaaagtt
```

```
           1        10        20        30     37
           |--------+---------+---------+------|
   LOSS12  TTTAAAGTCTA-TGTCACAGATTTTGAAATTCAGTGA
 LOSS13-2  ATTCAAGTTTT-AGTCTAAATAATTGTCTTTCAGTTA
 LOSS13-1   TTCAAGGCTACTGACCCTAAGAGTGACTATAAATTA
   LOSS10   TTTATCACTTTGTACATAAAAGTCGTCATGTAAATC
    LOSS8  GTGTATGTTTTTACAACTTGTGGAAAGATAAAAGAT
   LOSS16  TTTCATGTTTGGGTAATGGATGGAAGTTTTTGTTAA
 LOSS13-3  TTGTATATTAGTTAGTTTTCCAGCCTTTTTTAAAAG
    LOSS4  TTTTTGGTTTTCTTTGACTAAATTCTTTGTAATTTA
Consensus  tTttatgtttt...a...taaag..gt.tttaa.a..
```

```
           1        10        20        30       39
           |--------+---------+---------+--------|
   LOSS12  TTTAAAGTCTA-TGTCACAGATTTTGAAATTCAGTGA
 LOSS13-2  ATTCAAGTTTT-AGTCTAAATAATTGTCTTTCAGTTA
 LOSS13-1   TTCAAGGCTACTGACCCTAAGAGTGACTATAAATTA
   LOSS16  TTTCATGTTTG--GGTAATGGATGGAAGTTTTTGTTAA
 LOSS13-3   TTGTATATTA--GTTAGTTTTCCAGCCTTTTTTAAAAG
Consensus  .TTcaag.tTa..Gtta.t......gacttTtt.ttAa.
```

```
           1        10        20        30      38
           |--------+---------+---------+-------|
    LOSS8   GTGTATGTTTTTACAACTT-GTGGAAAGATAAAAGAT
   LOSS10  TTTATCACTTTGTACATAAAAGTCGTCATGTAAATC
    LOSS4  TTTTTGGTTTTCTTTGACTAAATTCT-TTGTAATTTA
Consensus  ttT.T...TTT.TacaactaagT.gt.atgTAAat.a.
```

Supplementary Fig. 16: ACS of various *L. thermotolerans* regions. **a.** LOGO generated using WEBLOGO (Crooks GE et al., Genome Research, 2004). **b.** Multiple alignment using multalin (Corpet, Nucl. Acids Res., 1988) of the ACS-containing DNA sequences used to generate the logo representations in a.

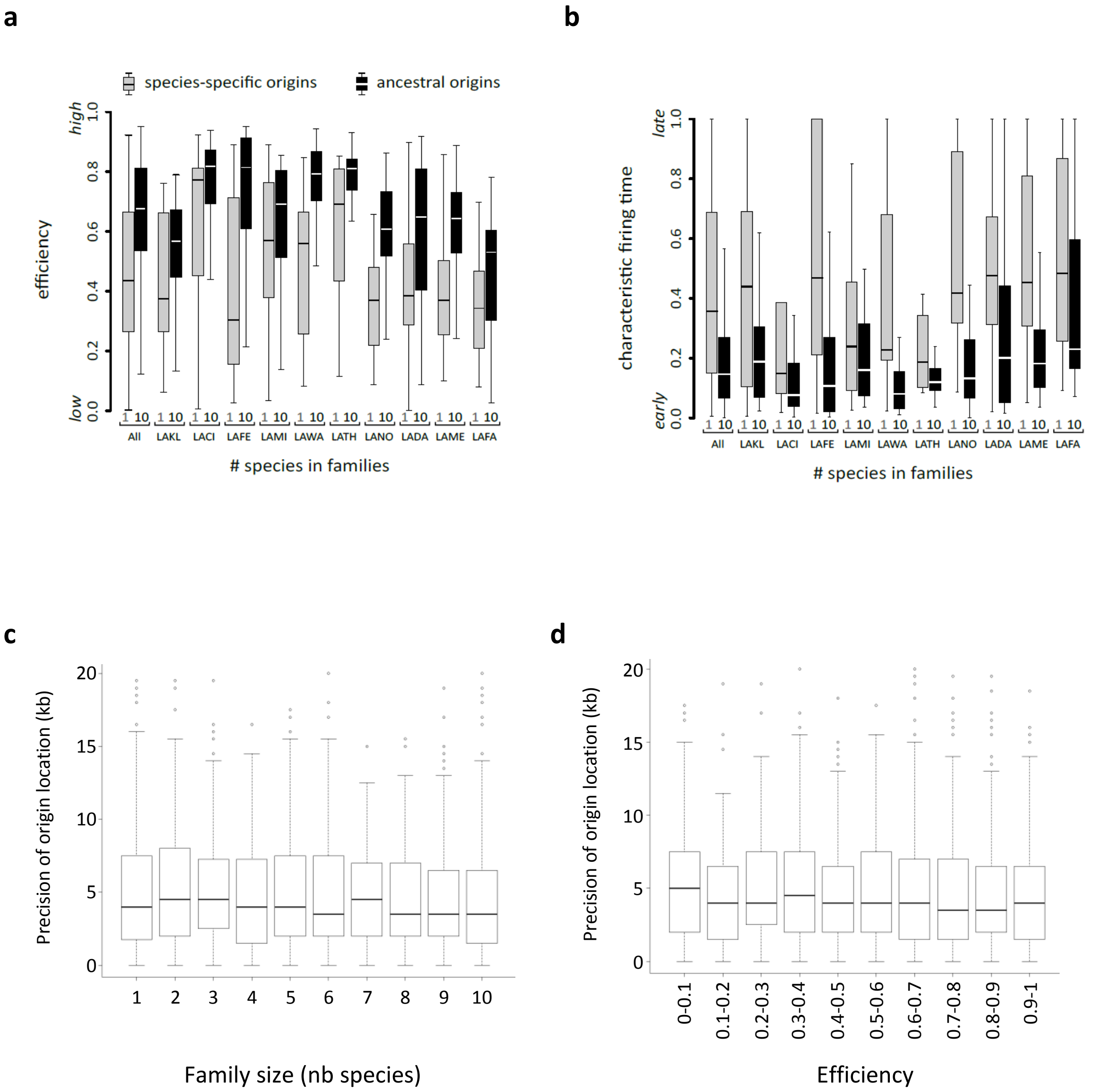

Supplementary Fig. 17: Relationship between functional properties of active replication origins and origin family size. Differences in efficiencies (**a**) and firing times (**b**) between species-specific origins (in grey) and ubiquitous origins shared by all 10 species (in black). Species names are abbreviated as in Fig. 3. We checked that these differences in efficiency and firing time between singleton and ubiquitous origins were not due to a methodological problem during the clustering of low efficiency origins. Given that the family clustering was solely based on chromosomal locations, we checked whether the precision of origin location was not linked to the family size (**c**) or affected by origin efficiency (**d**), ruling out the possibility that species-specific origins would have remained singletons due to their low efficiency. For all graphs, boxplot whiskers are defined by selecting the value within the range of the 75th and 25th percentile plus or minus 1.5 x (75th percentile – 25th percentile) respectively, and at the maximum distance from the median.

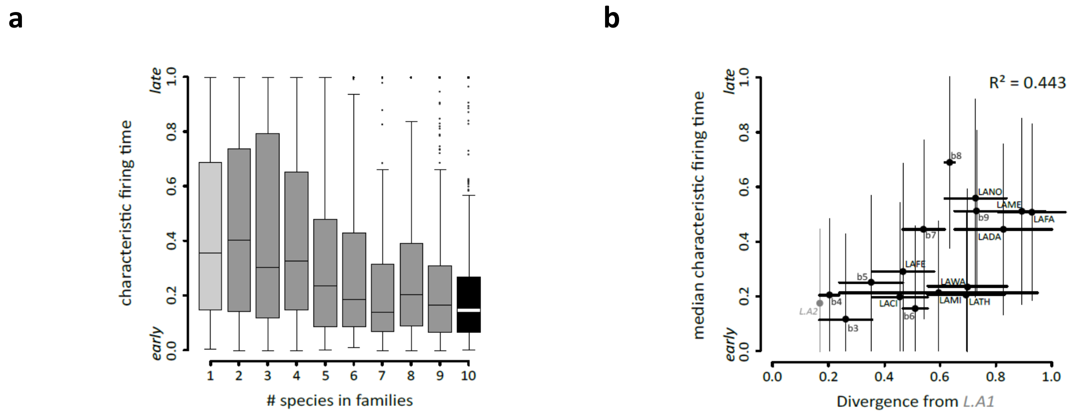

Supplementary Fig. 18: Relationship between characteristic firing times and origin evolutionary age. **a.** Distribution of the characteristic firing times as a function of the number of species comprised into the families of orthologous origins. Boxplot whiskers are defined by selecting the value within the range of the 75th and 25th percentile plus or minus 1.5 x (75th percentile – 25th percentile) respectively, and at the maximum distance from the median. **b.** Correlation between characteristic firing times and origin age for 546 origin families comprising 220 ancestral and 326 *Lachancea*-specific families. Each dot represents the median characteristic firing time of all the replication origins that appeared in a given branch of the phylogenetic tree. The x-axis represents the total branch length between *L.A2* (the last common ancestor of the 9 species indicated in light-grey) and the branch of appearance of the new origins. The most ancestral origins are on the left while the youngest lie on the right of the plot. Vertical error bars represent the standard deviation of origin efficiencies and horizontal bars represent the span of the branch lengths based on the phylogenetic tree in Fig. 3.

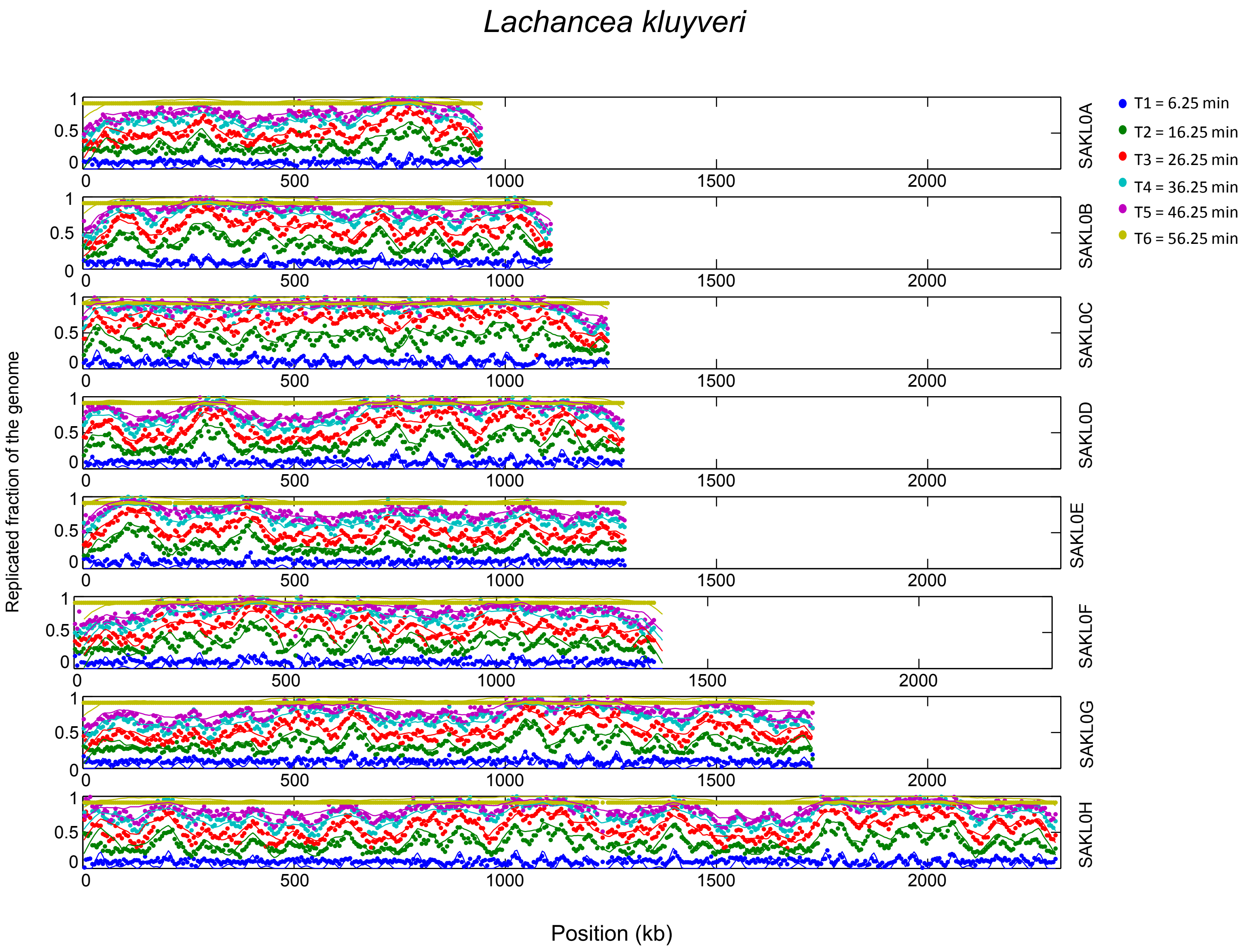

Lachancea kluyveri
Replicated fraction of the genome
Position (kb)
SAKL0A
SAKL0B
SAKL0C
SAKL0D
SAKL0E
SAKL0F
SAKL0G
SAKL0H
T1 = 6.25 min
T2 = 16.25 min
T3 = 26.25 min
T4 = 36.25 min
T5 = 46.25 min
T6 = 56.25 min

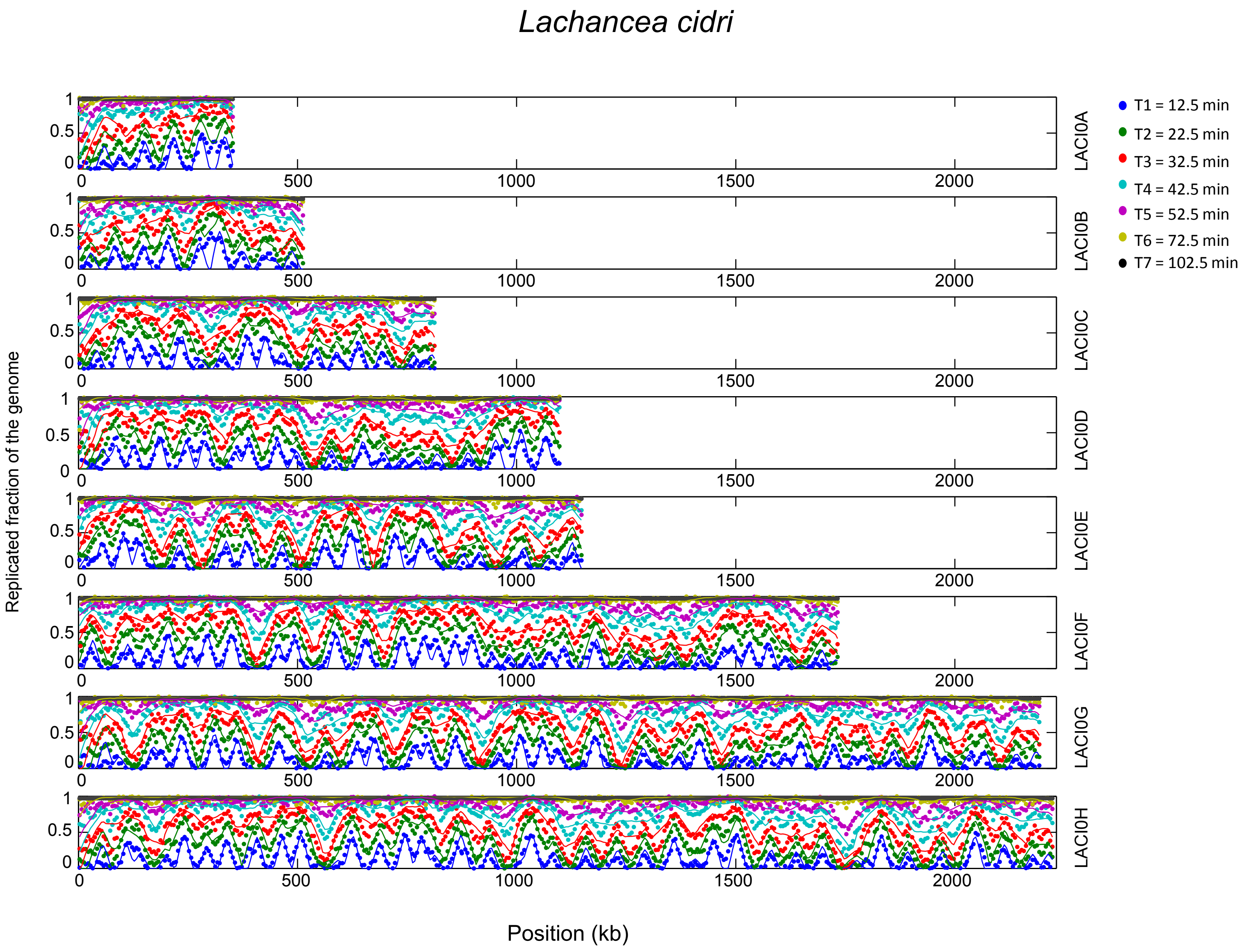
Lachancea cidri
Replicated fraction of the genome
Position (kb)
LACI0A
LACI0B
LACI0C
LACI0D
LACI0E
LACI0F
LACI0G
LACI0H
T1 = 12.5 min
T2 = 22.5 min
T3 = 32.5 min
T4 = 42.5 min
T5 = 52.5 min
T6 = 72.5 min
T7 = 102.5 min
0
0.5
1
500
1000
1500
2000

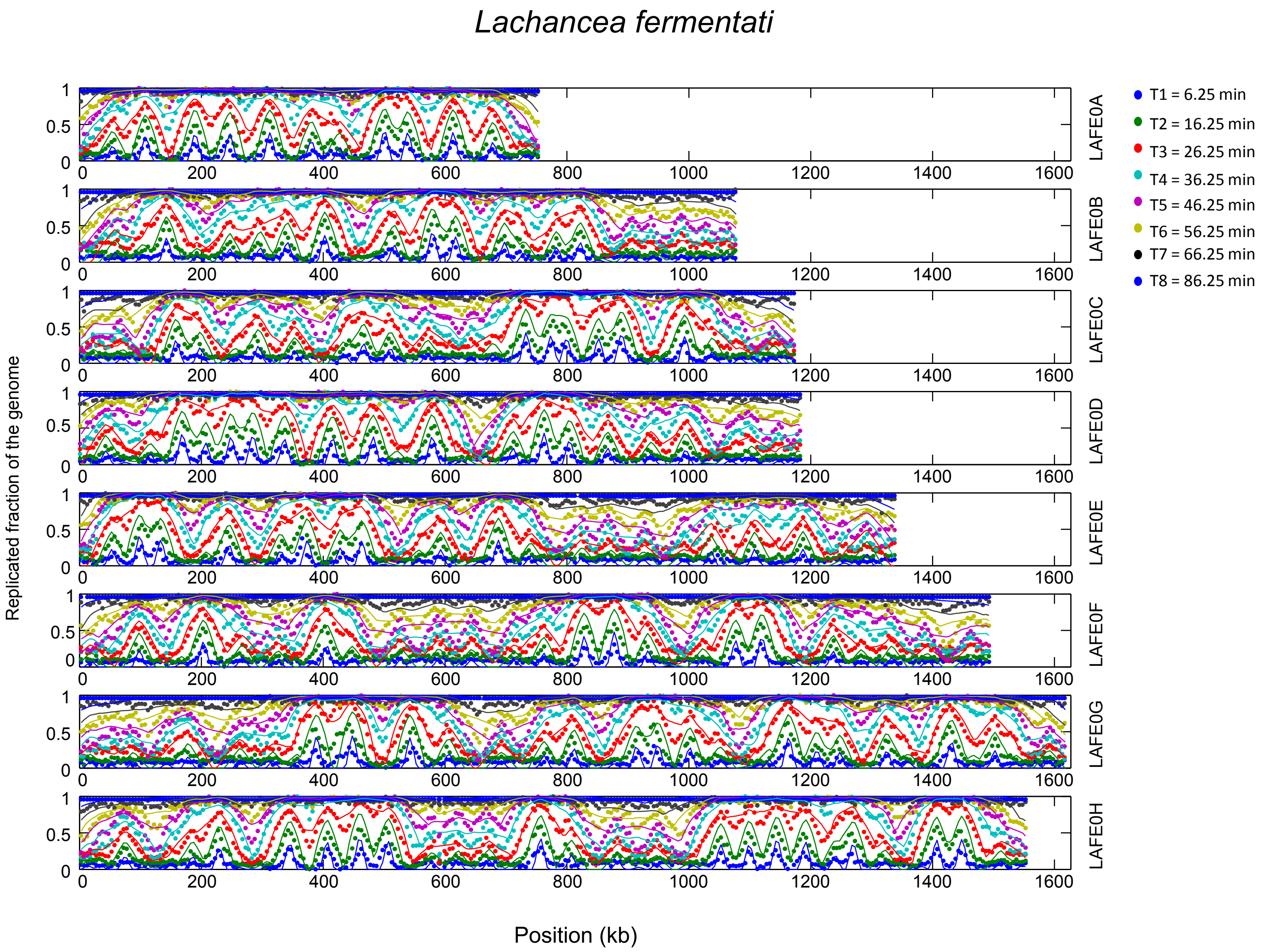
Lachancea fermentati
Replicated fraction of the genome
Position (kb)
LAFE0A
LAFE0B
LAFE0C
LAFE0D
LAFE0E
LAFE0F
LAFE0G
LAFE0H
T1 = 6.25 min
T2 = 16.25 min
T3 = 26.25 min
T4 = 36.25 min
T5 = 46.25 min
T6 = 56.25 min
T7 = 66.25 min
T8 = 86.25 min
0
200
400
600
800
1000
1200
1400
1600
0
0.5
1

Lachancea mirantina

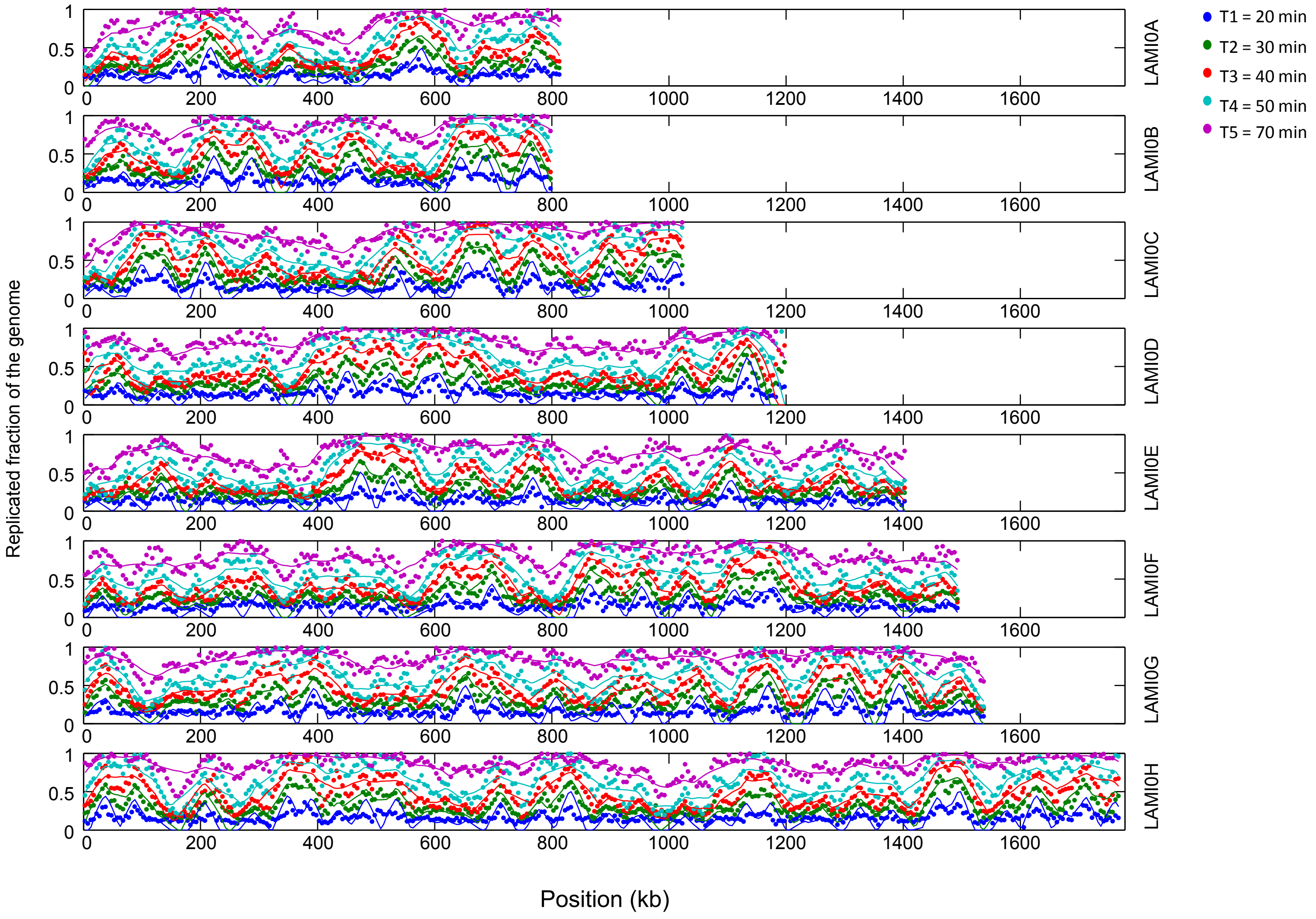

Lachancea waltii

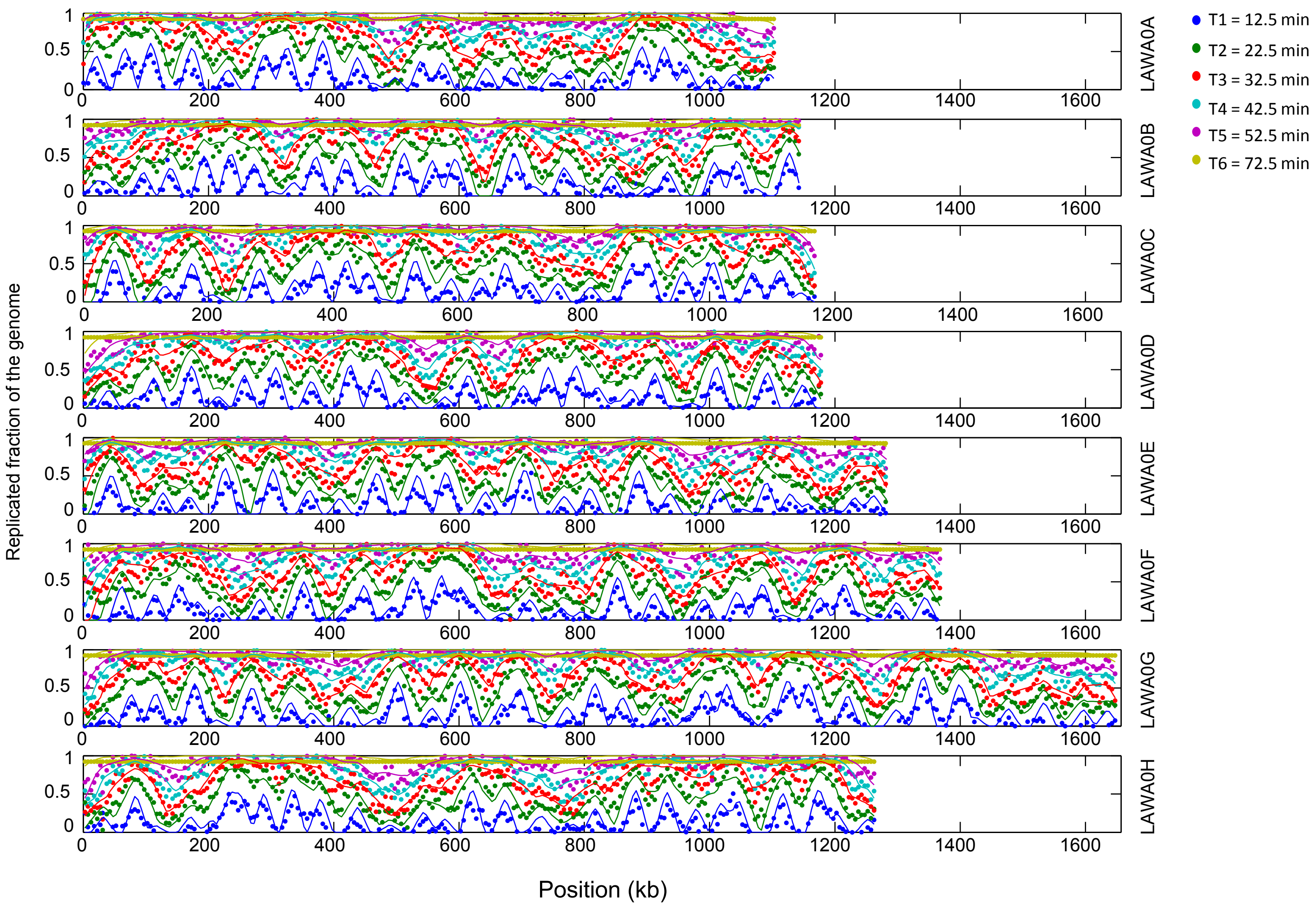

Lachancea thermotolerans

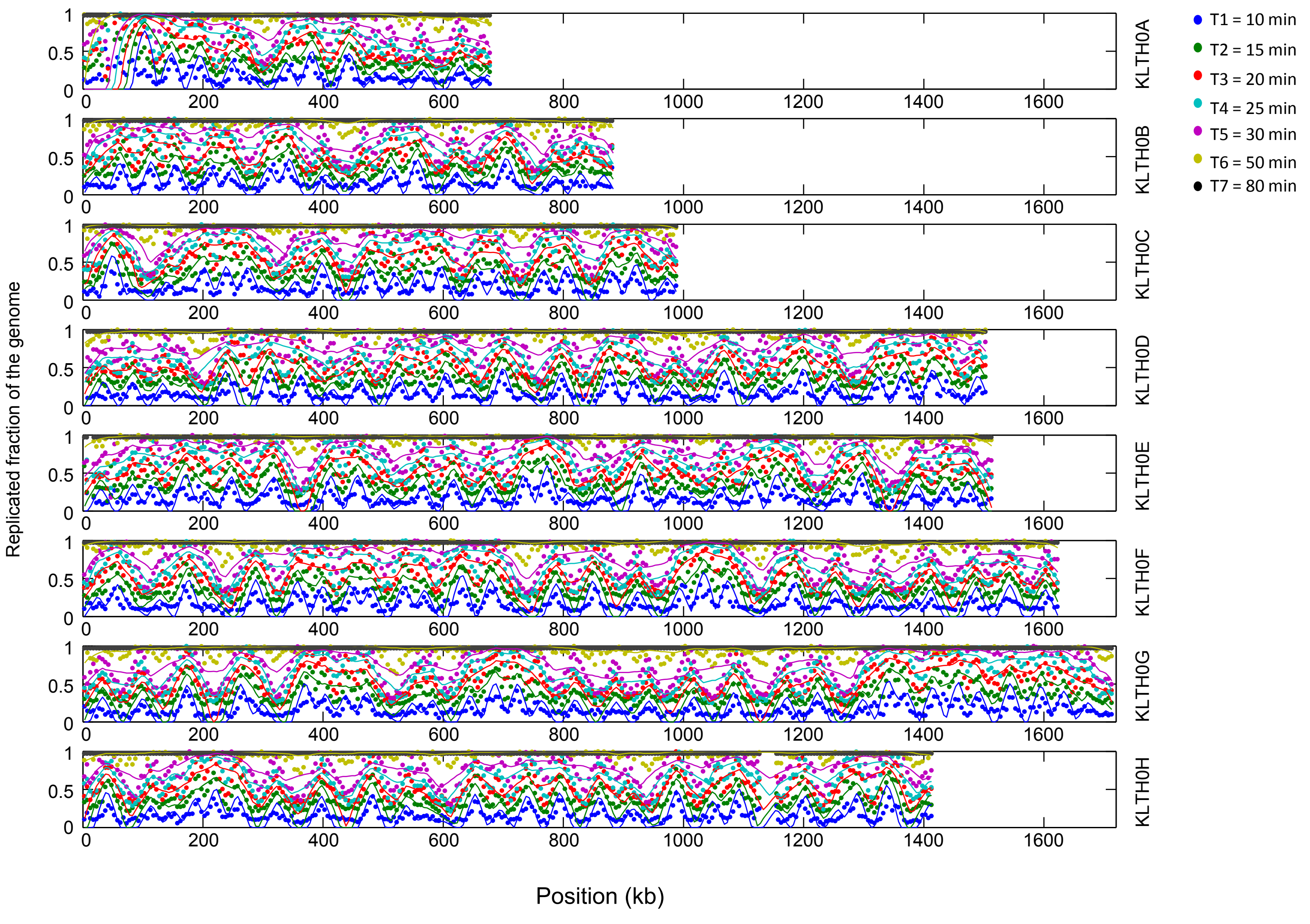

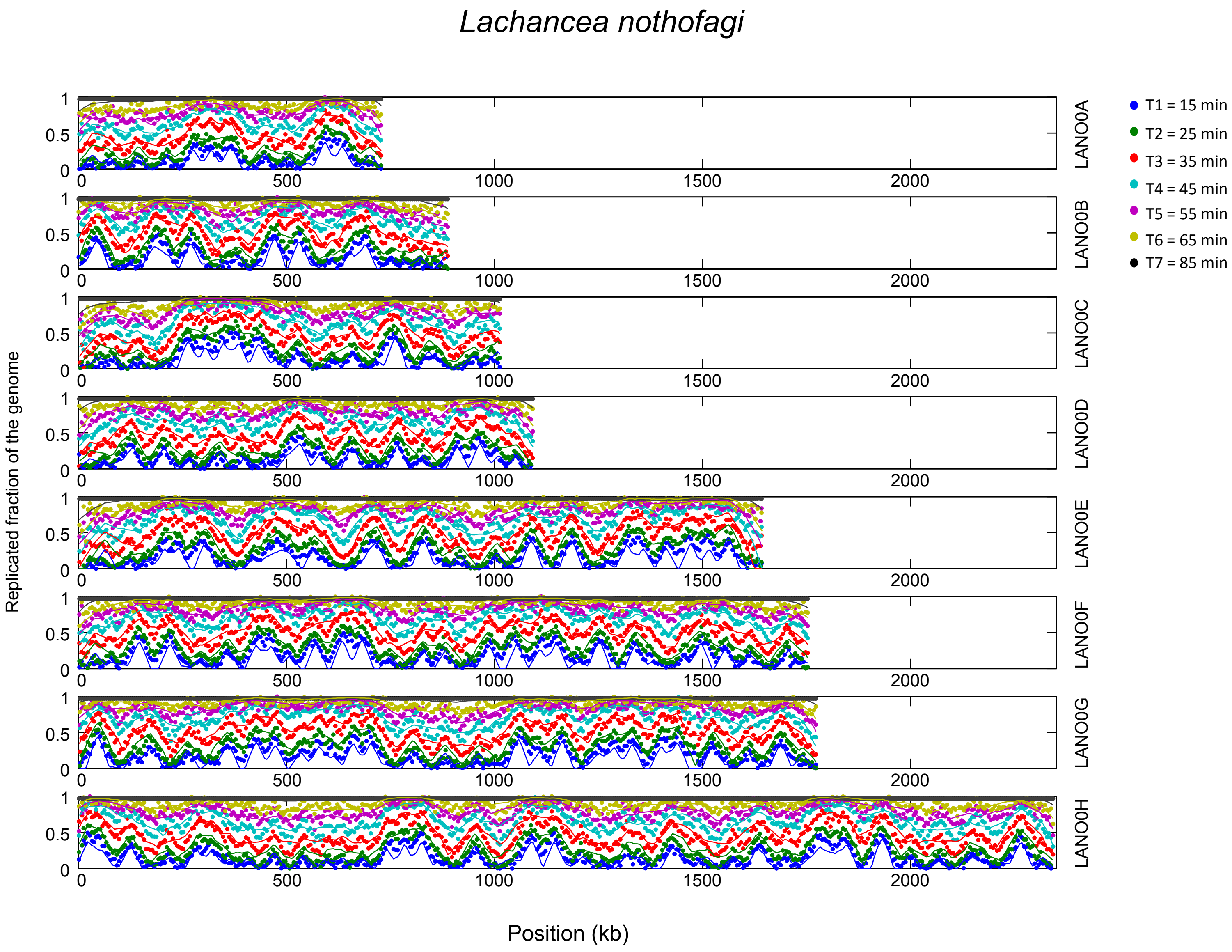

Lachancea nothofagi
Replicated fraction of the genome
Position (kb)
LANO0A
LANO0B
LANO0C
LANO0D
LANO0E
LANO0F
LANO0G
LANO0H
T1 = 15 min
T2 = 25 min
T3 = 35 min
T4 = 45 min
T5 = 55 min
T6 = 65 min
T7 = 85 min
0
500
1000
1500
2000
0.5
1

Lachancea dasiensis

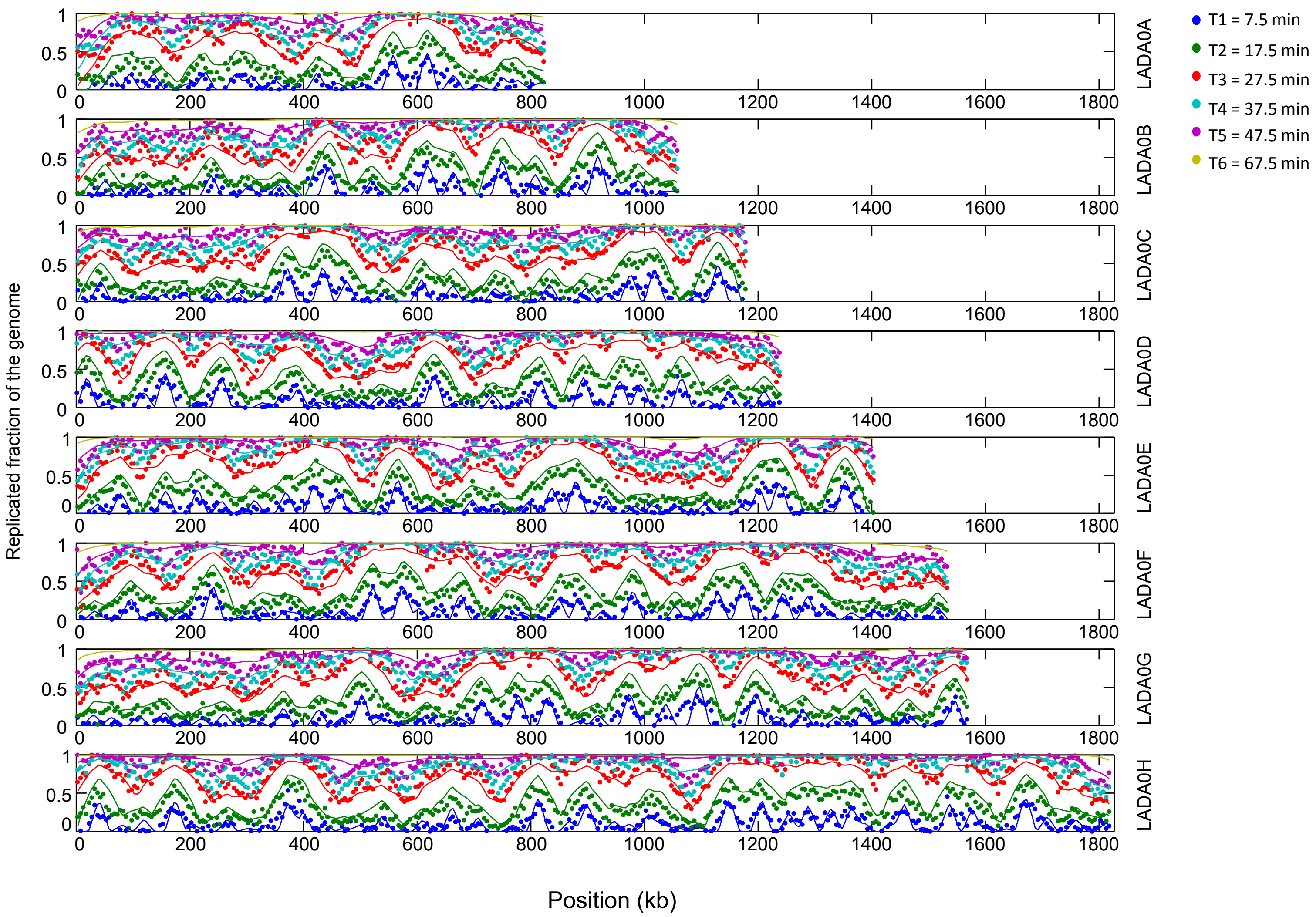

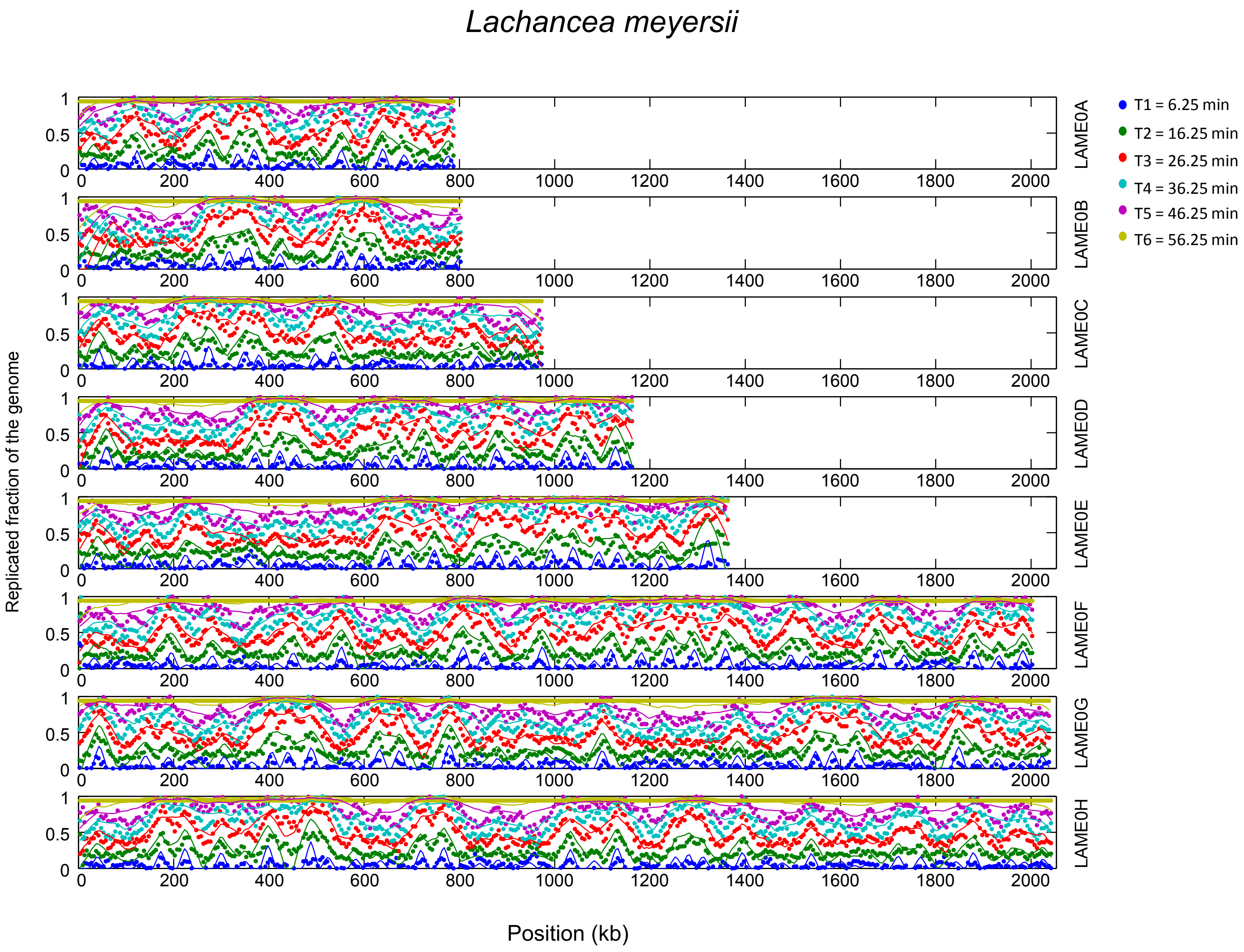

Lachancea meyersii
Replicated fraction of the genome
Position (kb)
LAME0A
LAME0B
LAME0C
LAME0D
LAME0E
LAME0F
LAME0G
LAME0H
T1 = 6.25 min
T2 = 16.25 min
T3 = 26.25 min
T4 = 36.25 min
T5 = 46.25 min
T6 = 56.25 min

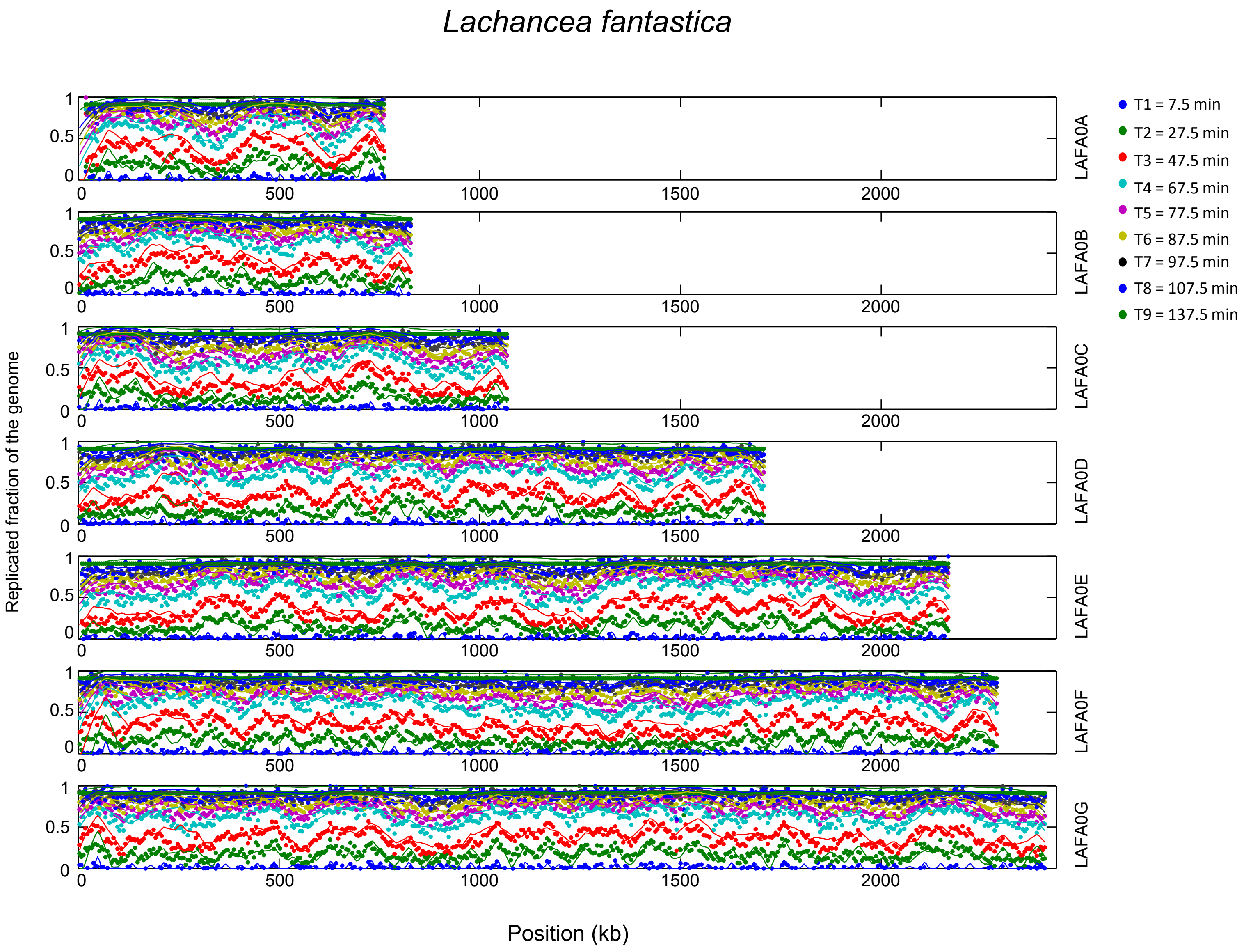

Lachancea fantastica
Replicated fraction of the genome
Position (kb)
LAFA0A
LAFA0B
LAFA0C
LAFA0D
LAFA0E
LAFA0F
LAFA0G
T1 = 7.5 min
T2 = 27.5 min
T3 = 47.5 min
T4 = 67.5 min
T5 = 77.5 min
T6 = 87.5 min
T7 = 97.5 min
T8 = 107.5 min
T9 = 137.5 min

Supplementary Fig. 19: Comparison of the experimental replication profiles at all time points with fits of the theoretical model. For every species, empirical data (symbols) and theoretical data (continuous lines of the same color of the symbols) are represented. Both data and model are averaged with a bin of 5Kb. Note that the empirical data were normalized between 0 (Start of the replication) and 1 (all the genome is replicated). Different colors indicate the different sampled time points from the beginning of S-phase.

Species	Strain	ploidy	mating type	growth media	growth temperature	Replication assay temperature
L. kluyveri	LAKL001	haploid	a	YPD	30°C	23°C
L. cidri	CBS4575	diploid	a/alpha	YPD	30°C	23°C
L. fermentati	CBS707	diploid	a/alpha	YPD	30°C	23°C
L. mirantina	CBS11717 (CLIB1160)	haploid	-	YPD	30°C	23°C
L. thermotolerans	CBS6340	diploid	a/alpha	YPD	30°C	23°C
L. waltii	CBS6430	diploid	a/alpha	YPD	30°C	23°C
L. nothofagi	CBS11611	haploid	-	YPD	24°C	23°C
L. dasiensis	CBS10888	haploid	-	YPD	30°C	23°C
L. meyersii	CBS8951	haploid	-	YPD	30°C	23°C
L. fantastica	CBS6924	haploid	-	YPD	24°C	23°C

Supplementary Table. 1: Yeast strains and growth conditions.

fragment ID	Oligo name	Sequence (5'->3')	Size (bp)	Bording Gene
C1	Cons1-For	tccgaagacacaatgactctaggggtatcgcctgtaactgtggcg	2497	KLTH0F16280g-KLTH0F16324g
	Cons1-Rev	gtcatgataataatggtttcttaggtcgagccatagcgaacacaa		
C2	Cons2-For	tccgaagacacaatgactctaggggaaatgcagggactcccatcg	474	KLTH0G01298g-KLTH0G01320g
	Cons2-Rev	gtcatgataataatggtttcttaggataatgctaccggcatcgct		
C3	Cons3-For	tccgaagacacaatgactctaggggcgtgctccgagataatggca	1491	KLTH0D05126g-KLTH0D05148g
	Cons3-Rev	gtcatgataataatggtttcttaggtcgccagctccttgttgata		
C4	Cons4-For	tccgaagacacaatgactctaggggtgctgtgcctgtctcttctg	534	KLTH0A06754g-KLTH0A06776g
	Cons4-Rev	gtcatgataataatggtttcttaggagcagccaggcctaaattcc		
L1	Loss1-For	tccgaagacacaatgactctaggggacgaccacaacgaagagcag	361	KLTH0C04818g-KLTH0C04840g
	Loss1-Rev	gtcatgataataatggtttcttaggcaagacagaagccaggtgtg		
L2	Loss2-For	tccgaagacacaatgactctagggggctccctttgagcataccgt	4592	KLTH0C07590g-KLTH0C07524g
	Loss2-Rev	gtcatgataataatggtttcttaggagaatacatccgtggcagca		
L3	Loss3-For	tccgaagacacaatgactctaggggatgtttttgccaatcttgac	3808	KLTH0C11748r-KLTH0C11814r
	Loss3-Rev	gtcatgataataatggtttcttaggattagatacatgtgggcgag		
L4	Loss4-For	tccgaagacacaatgactctaggggcctggagcagagagcaagtc	4457	KLTH0D00550g-KLTH0D00594g
	Loss4-Rev	gtcatgataataatggtttcttaggcacgaagttccgctcacatc		
L5	Loss5-For	tccgaagacacaatgactctaggggcgatggcgaggagctgctaa	2090	KLTH0D05918g-KLTH0D05962g
	Loss5-Rev	gtcatgataataatggtttcttaggatcatagagcacgccacttgc		
L6	Loss6-For	tccgaagacacaatgactctagggggcgagctttctgcaaatcgt	1997	KLTH0D12254g-KLTH0D12276g
	Loss6-Rev	gtcatgataataatggtttcttaggaagtcgaatggaaccgcact		
L7	Loss7-For	tccgaagacacaatgactctagggggccgagatctatttgtttgggc	518	KLTH0F11858g-KLTH0F11880g
	Loss7-Rev	gtcatgataataatggtttcttaggccgctgctggttttacaaaga		
L8	Loss8-For	tccgaagacacaatgactctaggggagatttggcgtgggcaagat	4253	KLTH0G07018g-KLTH0G06996g
	Loss8-Rev	gtcatgataataatggtttcttaggtcattcatgtcacaaattctgatgc		
L9	Loss9-For	tccgaagacacaatgactctaggggccaagaaaagacctgttcccc	1663	KLTH0B03234g
	Loss9-Rev	gtcatgataataatggtttcttaggatgtgaccctcgaaggattc		
L10	Loss10-For	tccgaagacacaatgactctaggggccccatgtttggcggatttc	4538	KLTH0F07480g
	Loss10-Rev	gtcatgataataatggtttcttaggcttagggaagtgtacgcggc		
L11	Loss11-For	tccgaagacacaatgactctaggggagcttgcccttttgcgagaa	2000	KLTH0H14564g-KLTH0H14586g
	Loss11-Rev	gtcatgataataatggtttcttaggtcccgaacactcggtctct		
L12	Loss12-For	tccgaagacacaatgactctaggggttgtttcttgatgcccacttgt	431	KLTH0H15290r-KLTH0H15312g
	Loss12-Rev	gtcatgataataatggtttcttaggcctacaaagatgaacgcggc		
pRS41 backbone	pRS41K-For	attttaataatttggatcagctgatgacccaggtggcacttttcg	4665	-
	pRS41K-Rev	cctaagaaaccattattatc		
LATH-CenC	CenC-For	atcagctgatccaaatta	128	-
	CenC-Rev	attgtgtcttcggaaccc		
TEST-Centro	TESTCenC-For	TTCATTCAGCTCCGGTTCCC	765	-
	TESTCenC-Rev	GTCTTCGGAACCCATATACAATTT		
TEST-ARS	TESTARS-For	AGGGTTATTGTCTCATGAGCGG	-	-
	TESTARS-Rev	CAGACAAGCTGTGACCGTCT		

Supplementary Table. 2: Oligonucleotides used to construct the ARS assay plasmids.
