# Supplementary material for: The evolution of the temporal program of genome replication
Source: Nat Commun. 2018 Jun 6;9:2199. doi: 10.1038/s41467-018-04628-4 (PMC5989221; doi:10.1038/s41467-018-04628-4)
Supplement: Supplementary file 3 — Description of Additional Supplementary Files [file 41467_2018_4628_MOESM3_ESM.pdf]

Description of Additional Supplementary Files

File Name: Supplementary Data 1
Description: Replication timing data. The spreadsheet entitled 'Origin location in ALL species' provides the chromosomal location as well as the efficiency the characteristic firing time of each replication origin in all 10 *Lachancea* species. The other 10 spreadsheets give the complete replication timing data for the 10 *Lachancea* species in non-overlapping windows of 500 bp. For both the time course experiment and the Marker Frequency Analaysis, the actual ratios namely ‘Trep’ and ‘Ratio Expo/Stat’ as well as the fitted data called ‘Mean Replication Time’ and ‘MFA’, respectively, are provided (see Methods).
